# Discovering single cannabidiol or synergistic antitumor effects of cannabidiol and cytokine-induced killer cells on non-small cell lung cancer cells

**DOI:** 10.3389/fimmu.2024.1268652

**Published:** 2024-03-14

**Authors:** Yutao Li, Amit Sharma, Michèle J. Hoffmann, Dirk Skowasch, Markus Essler, Hans Weiher, Ingo G. H. Schmidt-Wolf

**Affiliations:** ^1^Department of Integrated Oncology, Center for Integrated Oncology (CIO) Bonn, University Hospital Bonn, Bonn, Germany; ^2^Department of Neurosurgery, University Hospital Bonn, Bonn, Germany; ^3^Department of Urology, Medical Faculty, Heinrich Heine University Düsseldorf, Düsseldorf, Germany; ^4^Department of Internal Medicine II, Cardiology, Pneumology and Angiology, University Hospital Bonn, Bonn, Germany; ^5^Department of Nuclear Medicine, University Hospital Bonn, Bonn, Germany; ^6^Department of Applied Natural Sciences, Bonn-Rhein-Sieg University of Applied Sciences, Rheinbach, Germany

**Keywords:** cytokine-induced killer cells, cannabidiol, immunotherapy, transient receptor potential vanilloid Type 2, long interspersed nuclear element-1, DNA methylation, DNA double-strand breaks

## Abstract

**Introduction:**

A multitude of findings from cell cultures and animal studies are available to support the anti-cancer properties of cannabidiol (CBD). Since CBD acts on multiple molecular targets, its clinical adaptation, especially in combination with cancer immunotherapy regimen remains a serious concern.

**Methods:**

Considering this, we extensively studied the effect of CBD on the cytokine-induced killer (CIK) cell immunotherapy approach using multiple non-small cell lung cancer (NSCLC) cells harboring diverse genotypes.

**Results:**

Our analysis showed that, a) The Transient Receptor Potential Cation Channel Subfamily V Member 2 (TRPV2) channel was intracellularly expressed both in NSCLC cells and CIK cells. b) A synergistic effect of CIK combined with CBD, resulted in a significant increase in tumor lysis and Interferon gamma (IFN-g) production. c) CBD had a preference to elevate the CD25+CD69+ population and the CD62L_CD45RA+terminal effector memory (EMRA) population in NKT-CIK cells, suggesting early-stage activation and effector memory differentiation in CD3+CD56+ CIK cells. Of interest, we observed that CBD enhanced the calcium influx, which was mediated by the TRPV2 channel and elevated phosphor-Extracellular signal-Regulated Kinase (p-ERK) expression directly in CIK cells, whereas ERK selective inhibitor FR180204 inhibited the increasing cytotoxic CIK ability induced by CBD. Further examinations revealed that CBD induced DNA double-strand breaks via upregulation of histone H2AX phosphorylation in NSCLC cells and the migration and invasion ability of NSCLC cells suppressed by CBD were rescued using the TRPV2 antagonist (Tranilast) in the absence of CIK cells. We further investigated the epigenetic effects of this synergy and found that adding CBD to CIK cells decreased the Long Interspersed Nuclear Element-1 (LINE-1) mRNA expression and the global DNA methylation level in NSCLC cells carrying KRAS mutation. We further investigated the epigenetic effects of this synergy and found that adding CBD to CIK cells decreased the Long Interspersed Nuclear Element-1 (LINE-1) mRNA expression and the global DNA methylation level in NSCLC cells carrying KRAS mutation.

**Conclusions:**

Taken together, CBD holds a great potential for treating NSCLC with CIK cell immunotherapy. In addition, we utilized NSCLC with different driver mutations to investigate the efficacy of CBD. Our findings might provide evidence for CBD-personized treatment with NSCLC patients.

## Introduction

Undoubtedly, cannabidiol (CBD) can relieve cancer pain and even mitigate the side effects of chemotherapy, yet there has been a paucity of knowledge concerning its anti-cancer effect. It has been discussed for decades that the activation of cannabinoid receptors (CB1 and CB2) may exert anti-inflammatory, pro-apoptotic and anti-proliferative effects that could help fight cancer (1, 2). Besides, the receptors (G-protein receptors; transient receptor potential vanilloid ion channels (TRPV1 and 2); peroxisome proliferator-activated receptors) that CBD interacts with, are also believed to be involved in several key biological processes (2, 3). Cannabinoid receptors (CB1 and CB2 receptors) are not the ligands of CBD. CBD has low affinity for CB1 and CB2 receptors (4). A previous study on nanomolar-range high affinity binding demonstrated a negative effect of CBD on cannabinoid receptor activation through an allosteric site (5). However, CBD acts as a selective TRPV2 agonist. CBD preferentially binds to TRPV2 rather than TRPV1 based on computational and structural data (6). Therefore, we focus on the TRPV2 channel in this study. In preclinical models, like other cancer types, CBD-related research on lung cancer (LC) has also been conducted. LC is known to involve an accumulation of genetic and epigenetic events and, with its two main subtypes (small cell lung cancer/SCLC and non-small cell lung cancer/NSCLC), is responsible for numerous cancer-related deaths (7–9). Recently, it was shown that CBD decreased viability and induced cell death in lung cancer stem cells and adherent lung cancer cells (10). The authors also revealed that CBD activated the effector caspases 3/7, increased the expression of pro-apoptotic proteins and levels of reactive oxygen species. An independent study showed CBD as a novel therapeutic agent targeting TRPV2 to inhibit the growth and metastasis of aggressive cisplatin-resistant NSCLC (11). CBDs have been shown to increase the lysis of lung cancer cells by lymphokine-activated killer cells via upregulation of Intercellular Adhesion Molecule 1 (ICAM-1) (12). Additionally, it has been demonstrated that CBD can inhibit cancer-associated fibroblasts (13) and suppress proliferation and reduce *in vitro* migration of lung cancer cell lines (14). Mechanically, a combination of THC (Tetrahydrocannabinol) and CBD leads to the signaling activation of mitogen-activated protein kinase pathway (ERK1/2 phosphorylation) in HEK 293 cells (15).

The TRPV2 channel is implicated in signaling pathways that mediate cell survival, proliferation, and metastasis. TRPV2 is a homotetrameric N-glycosylated protein that is largely located in the endoplasmic reticulum compartment under unstimulated conditions. When TRPV2 is stimulated by CBD, the activity of phosphatidylinositol 3-kinase (PI3K) triggers the translocation of TRPV2 to the plasma membrane, where it acts as an ion channel. Subsequently, the increase in TRPV2-mediated Ca^2+^ entry leads to the activation of signaling pathways involved in cell survival, apoptosis, proliferation, differentiation, and metastasis (16). Previous studies demonstrated that TRPV1 was functionally expressed in CD4^+^ T cells, contributed to T cell antigen receptor (TCR)-induced Ca^2+^ influx, TCR signaling and T cell activation (17). However, there are no investigations indicating that TRPV2 impacts T cell activation.

KRAS oncogenes are the most commonly mutated oncogenes in non-small cell lung cancer, but effective therapeutic strategies to target RAS-mutant cancers have proved elusive since direct inhibition of RAS proteins has proved difficult (18). In a previous study, CBD induced cancer cell apoptosis via activation of p53-dependent apoptotic pathways in cancer cells with wild-type p53 (19, 20). A549 cells (KRAS-G12D mutation, P53 wide-type), NCI-H2228 (EML4-ALK variant 3, P53 Q331* mutation) and HCC-827 (EGFR exon19 deletion mutant, inactivation P53 mutation) used in the current study contain rearrangements of chromosomes and substantial changes in the genomic landscape. Besides, in our previous study, it was shown that NSCLC cells harboring an EML4-ALK rearrangement have higher responses to CIK therapy (21). In addition, Long Interspersed Nuclear Element-1 (LINE-1) promotes tumorigenicity and exacerbates tumor progression in NSCLC cell lines and mice model (22). A recent study reported that LINE-1 regulates T-cell quiescence and exhaustion (23). Owing to these findings, herein, we questioned: 1) whether CBD has any particular effects on LC cell lines with diverse genetic backgrounds; 2) whether CBD can exert any influence on CIK cells aiming to enhance their therapeutic potential or function targeting NSCLC cells. Of importance, we, for the first time in the literature, investigated if CBD or CBD in the presence of a lower amount of CIK cells can affect the expression and methylation of LINE-1 repetitive sequences in NSCLC cells.

## Materials and methods

### Regents and antibodies

CBD (purity≥98% (HPLC) (Bio-Techne, Wiesbaden-Nordenstadt, Germany) and tranilast (Cayman Chemical Company, Hamburg, Germany) were dissolved in dimethyl sulfoxide (DMSO). All antibodies used in the study such as fluorochrome-conjugated FITC anti-human CD3 antibody (clone OKT3), APC anti-human CD3 antibody (clone OKT3), PerCP/cyanine5. 5 anti-human CD3 antibody (clone OKT3), Brilliant Violet 421 anti-human CD8 antibody (clone RPA-T8), APC/cyanine7 anti-human CD4 antibody (clone RPA-T4), PE anti-human CD56 antibody (clone 5. 1 H11), APC anti-human CD56 antibody (clone 5. 1 H11), PE/Cyanine7 anti-human CD69 antibody (clone FN50), PE anti-human CD25 antibody (clone BC96), PE anti-human CD45RA antibody (clone HI100), FITC anti-human CD62L antibody (clone DREG-56) were purchased from Biolegend (San Diego, CA, U.S.A.).

### Cell culture

Cytokine-induce killer (CIK) cells were generated, as previously described (24, 25). PBMCs required for the experiments were isolated from the blood of healthy donors registered at the blood bank of University Hospital Bonn. Three human non-small cell lung cancer cell lines with distinct genotypes were used in this study: A549 cells (KRAS mutation, P53 wide-type), NCI-H2228 (EML4-ALK variant 3, P53 ^Q331*^ mutation) and HCC-827 (EGFR exon19 deletion mutant, inactivation P53 mutation). A549 and HCC-827 were purchased from Leibniz Institute DSMZ-German Collection of Microorganisms and Cell Cultures GmbH (DSMZ, Braunschweig, Leibniz, Germany) and NCI-H2228 was purchased from the American Type Culture Collection (ATCC, Manassas, VA, U.S.A.). All cell lines were tested for mycoplasma negative and cultured in RPMI 1640 medium supplemented with 10% heat-inactivated FBS (Gibco, Munich, Germany) and 1% penicillin/streptomycin (P/S) (Gibco, Munich, Germany) at 37°C (5% CO_2_).

### RNA extraction, reverse transcription (RT)-PCR and global LINE-1 methylation

RNA was isolated from the cells using the miRNeasy Micro kit (Qiagen, Hilden, Germany) and subsequently cDNA synthesis was performed using the SuperScript™ III First-Strand Synthesis Super Mix kit (Invitrogen, CA, U.S.A.), following the manufacturer’s instructions. QuantStadio 3 Real-Time PCR System (Applied Biosystems, CA, U.S.A.) using PowerTrack™ SYBR Green Master Mix (Applied Biosystems, CA, U.S.A.) was used for MMP-9 mRNA detection. QuantiTect SYBR^®^ Green PCR Kit (QIAGEN, Hilden, Germany) was used for LINE-1 mRNA measurement. The relative amount of target genes was normalized by the amount of HPRT for MMP-9. The primer sequences were: MMP-9-PF/MMP-9-PR (5`-CGC AGA CAT CGT CAT CCA GT-3`/5`-AAC CGA GTT GGA ACC ACG AC-3`) and HPRT-PF/HPRT-PR (5´-TCA GGC AGT ATA ATC CAA AGA TGG T-3´/5´-AGT CTG GCT TAT ATC CAA CAC TTC G-3´), as previously described (26). TATA binding protein (TBP) was used as the reference gene for LINE-1. The primer pairs for the amplification of LINE-1 and TBP were LINE-1-PF/LINE-1-PR (5′-GTA CCG GGT TCA TCT CAC TAG G-3′/5′-TGT GGG ATA TAG TCT CGT GGT G-3′), TBP-PF/TBP-PR (5′-ACA ACA GCC TGC CAC CTT A-3′/5′-GAA TAG GCT GTG GGG TCA GT-3′), as previously described (27). The experiments were repeated three times, and each sample was analyzed in triplicate by ΔΔCt method. For investigating global LINE-1 methylation, first genomic DNA was extracted using the Qiagen genomic DNA purification kit (Qiagen, Hilden, Germany), according to the manufacturer’s recommendations. Subsequently, the Global DNA Methylation LINE-1 kit (Active Motif, Carlsbad, CA, U.S.A.) was used to quantify global LINE-1 methylation levels. For synergistic experiments with a combination of CBD and CIK targeting NSCLC cells, mixture cells were washed with cold DPBS three times to exclude CIK cells, then only adherent NSCLC cells were on the six-well plate. NSCLC cells were trypsinized and washed twice with 2 mL ice-cold DPBS and cell pellets were finally harvested.

### Cell viability assessment by CCK-8 assay and the cytotoxicity of CIK cells analysis by flow cytometry

CCK-8 cell viability assay was performed, as described by the manufacturer (Dojindo Laboratories, Kumamoto, Japan). Briefly, NSCLC cells were seeded into 96-well plates (1x10^4^ cells/well) and treated with various concentrations of CBD or DMSO control for 24 h. Similarly, flow cytometry-based cytotoxicity was performed, as described protocol. Briefly, the target cells were labeled with CFSE (1 x 10^6^ cells in 1 ml PBS with 0.5 µM CFSE, 20 min, 37°C in the dark) and washed twice with warm culture medium. CFSE-labeled 5 x 10^4^ tumor cells were incubated at various concentrations of CBD for 24 h with CIK cells to perform redirected cytolysis assay at an E:T ratio of 10:1. Following 24 h of culturing, the cells were stained with Hoechst 33258 (Cayman Chemical, Hamburg, Germany) and were quantified using BD FACS Canto II. Then the percentage of cytotoxicity of CIK cells was analyzed by FlowJo V10 software (Tree Star, Ashland, Oregon) and calculated by the following formula as previously described (25):


Specific lysis (%)=((CT − TE)/CT) × 100

CT: cell count of live CFSE^+^ tumor cells in control tubes (tumor cells alone); TE: cell count of live CFSE^+^ tumor cells in test tubes (tumor + effector or tumor + CBD).

To dissect the underlying mechanisms of the synergistic effects of the combination treatment, we have investigated whether combination-induced cytotoxicity was related to inhibition of ERK signaling pathways. CIK cells were incubated with 10 µM ERK-selective inhibitor FR180204 (Cayman Chemical, Hamburg, Germany) for 2 h before being cocultured with CFSE-labeled tumor cells at various concentrations of CBD for 24 h, as previously described (28, 29).

### Enzyme-linked immunosorbent assay (ELISA)

The ELISA assay was performed using the standard protocol. Briefly, 5 x 10^5^ CIK cells were co-cultured with 5 x 10^4^ tumor cells in the presence of various concentrations of CBD or DMSO control for 24 h at E:T 10:1. Thereafter, the cell-free supernatant was collected to perform sandwich ELISA assay (IFN Gamma Kit, Invitrogen, Camarillo, CA, U.S.A.), according to the manufacturer’s instructions.

### Intracellular calcium response of CIK cells and intracellular expression of P-ERK and TRPV2 by flow cytometry

1x10^6^ CIK cells were stained with 1 µM Fluo-4 AM (Thermo Fisher Scientific, Waltham, U.S.A.) in DPBS (with calcium and magnesium) for 20 minutes at 37°C. Cells were pelleted and resuspended in DPBS (with calcium and magnesium) and were subsequently exposed to CBD/TRPV2 antagonist tranilast (TLS) at 37°C for 1 minute. Dead cells were gated and excluded by Hoechst 33258 for Fluo-4 AM expression. Cells were acquired for 30 seconds and analyzed on an BD FACS Canto II on the FITC channel. To determine the intracellular p-ERK expression, a Fixable Viability Zombie Aqua™ Dye was used to exclude dead cells from the analysis. Mainly, CIK cells were incubated with Brefeldin A, which is a protein transport inhibitor commonly used to enhance intracellular staining signals. After gently mixing, the cells were then incubated with CBD for 15 minutes and then fixed with 100 µL fixation buffer for 30 min at room temperature in the dark. Cells were then washed and resuspended in 100 µL 1x True-Phos™ Perm Buffer at -20°C for 1 h and then stained with FITC anti-ERK1/2 Phospho (Thr202/Tyr204) antibody for 30 min at room temperature. Subsequently, the cells were washed twice with 2 mL DPBS, centrifuged at 1000 x g at room temperature for 5 minutes, and recorded with a BD Canto II cytometer. All reagents were purchased from Biolegend (San Diego, CA, U.S.A.). Since not all cell surface markers are compatible with BioLegend’s True-Phos™ Perm Buffer, the subpopulations of CIK cells were not further identified in this experiment.

CIK cells or NSCLC cells were stained with a Fixable Viability Zombie Aqua™ Dye exclude dead cells. After that, CIK cells were stained by APC anti-human CD3 antibody (clone OKT3), Brilliant Violet 421 anti-human CD8 antibody (clone RPA-T8), APC/cyanine7 anti-human CD4 antibody (clone RPA-T4), PE anti-human CD56 antibody (clone 5. 1 H11) at 4°C for 20 min. Afterwards, the cells were fixed and permeabilized using fixation buffer and intracellular staining permeabilization wash buffer (Biolegend, San Diego, CA, U.S.A.). The blocking step was carried out in permeabilization wash buffer with 2% normal goat serum for 15 min. Cells were incubated with anti-TRPV2 antibody (1:100 dilution, EpigenTek, Farmingdale, NY, U.S.A) at 4°C for 30 min. Subsequently, cells were washed and incubated with Alexa Fluor™ 488-labeled goat anti-rabbit IgG (H+L) (1:250 dilution, A-11008, Invitrogen, CA, U.S.A.) for 30 min. After staining, cells were washed and suspended in DPBS for flow cytometric analysis, as previously described (30).

### Immunocytochemistry (ICC) for detection of phospho-γH2AX/TRPV2

NSCLC cells were seeded on the sterile round coverslips Ø13mm (Carl Roth GmbH + Co. KG, Karlsruhe, Germany) with 0.1% gelatin coating on a 24-well plate at a density of 5 × 10^3^ cells/well in complete culture medium. After 48 h, cells were incubated with 10 µM CBD or 10 µM CBD with 10 µM TLS for 3 h in fresh culture medium. We observed that approximately half of A549 cells lost the capability of adhesion and climbing cover slides at 15 µM and 20 µM concentrations of CBD. Epithelial adhesion to cover slides is necessary in confocal experiments. Therefore, we assessed p-γH2AX expression immunofluorescence at 10 µM cannabidiol. The cells were fixed in 4% paraformaldehyde (PFA) and permeabilized with 0.1% Triton in PBS for 10 min. Alternatively, 1 × 10^6^ cells/well CIK cells were incubated with 10 µM CBD or DMSO for 3 h in fresh CIK culture medium. Fix the cells with 4% PFA for 20 min by adding an equal volume of 4% PFA to the culture medium. After that, the cells were resuspended in 200 μL deionized H_2_O. Add 5 uL cell suspension to 0.1% gelatin-coated slide (3 spots per slide) and smear with the side of a pipette tip. Air dry to evaporate for 2 min. The subsequent steps are the same as for NSCLC cells. Cells were then washed three times in PBS with 0.1% BSA. The blocking step was carried out in PBS with 10% normal goat serum and 0.3% Triton for 45 min. Thereafter, the anti-phospho-Histone H2AX (Ser139) antibody (clone JBW301) (Merck Millipore, Darmstadt, Germany) was diluted 1:500, and secondary antibody Alexa Fluor 488 (Thermo Fisher Scientific, San Diego, CA, U.S.A.) was diluted 1:2000 in dilution buffer (1% BSA, 0.3% Triton in PBS). Finally, diluted DAPI solution was added to each slice. Morphological analysis was performed by Visitron VisiScope Spinning Disk Confocal Microscopy (Visitron Systems GmbH, Puchheim, Germany). Confocal images were acquired using VisiVIEW^®^ Image software. The image analyses were facilitated by FiJi ImageJ software (31). In order to detect TRPV2 expression, the anti-TRPV2 polyclonal rabbit antibody (EpigenTek, Farmingdale, NY, U.S.A) was diluted 1:200, and the secondary antibody Donkey Anti-Rabbit IgG NorthernLights™ NL557-conjugated antibody (R&D Systems, Inc., Minneapolis, Minnesota, U.S.A.) was diluted 1:500 in dilution buffer (1% BSA, 0.3% Triton in PBS).The slide was finally stained with a diluted DAPI solution.

### Cell migration, invasion and wound healing measurements

The migration assay was conducted using a Cell Culture Insert with a pore size of 8 µm (BD Biosciences, Bedford, MA, U.S.A.) as previously described (32). Matrigel LDEV-Free Reduced Growth Factor Basement Membrane Matrix (Thermo Fisher Scientific, San Diego, CA, U.S.A.) was used to evaluate cell invasion potential. 200 uL of a 1:50 dilution of Geltrex matrix was added to each insert well and kept in a 37°C incubator for 1 h initially. 1 × 10^5^ tumor cells per well were seeded in the upper chamber in 200 µL serum-free RPMI 1640 medium with either 10 µM CBD, DMSO control or 10 µM CBD with 10 µM TRPV2 antagonist tranilast for 72 h. The lower chamber contained RPMI 1640 medium with 20% FBS to form a chemoattractant gradient to favor migration directionality. The chambers were incubated at 37°C for 96 h in 5% CO_2_, and non-migrating or non-invading cells were then removed from the upper side of the membrane. The migrating or invading cells in the lower chamber were washed with DPBS and fixed with 4% formaldehyde for 10 minutes. After 100% methanol permeabilizing for 15 min, the cells were stained with crystal violet 0.1% diluted in water (Sigma-Aldrich Chemie GmbH, Taufkirchen, Germany). Each assay was performed in triplicate. The migratory cells or invasive cells were observed and counted manually under light microscopy in six different fields.

The wound healing assay was performed as previously described (33–35), with slight modifications. Mainly, we utilized a sterile marker pen and a sterile ruler to draw four horizontal lines on the outside bottom of a 6-well plate. For preparing confluent monolayers, 2 x 10^5^ tumor cells were seeded and cultured to confluence 90% in six-well culture plates. Then, tumor cells in the culture medium were scratched to produce two ‘wound’ vertical lines using a sterile 20 µL pipette tip and a sterile ruler. Cell debris was removed from the culture by gently washing with sterile warm DPBS twice. The cells were then cultured in RPMI 1640 medium with 5% FBS in the presence of either 10 µM CBD or DMSO or 10 µM CBD combined with 10 µM tranilast for 24-72 h. The cell migration images in 15 different fields divided by horizontal and vertical lines were observed and acquired under 10X magnification by a Zeiss Primovert inverted cell culture microscope equipped with an Axio digital camera (Carl Zeiss, Oberkochen, Germany). Wound closure was monitored at 24-72 h, until the borders of the wound could no longer be identified. Images were compared with the same field at different timepoints and quantified using Fiji Image J software and the migration rate was calculated as the percentage of area reduction or wound closure as previously described (34).


$$\text{Wound Closure }\%=\;\;\left[\frac{At=0h\;-\;At=\Delta h}{At=0h}\right]$$

*A_t_*_=_*_0h_* is the area of the wound measured immediately after scratching (*t* = 0 h).

*A_t=_Δ_h_* is the area of the wound measured h hours after the scratch is performed.

### Genomic DNA extraction and global DNA methylation LINE-1 detection

Genomic DNA was extracted using the Qiagen genomic DNA purification kit (Qiagen, Hilden, Germany), according to the manufacturer’s recommendations. 1x10^6^ cells were lysed in lysis solution, after that QIAGEN protease was added, and the mixture was incubated at 50°C for 60 min. The samples were proceeded with Genomic-tip 20/G, washed and eluted. The genomic DNA was precipitated with isopropanol and washed with cold 70% ethanol. The DNA was dissolved in 10 mM Tris.HCL, PH 8.5) at 55°C overnight and quantified using a NanoDrop ND-1000 spectrophotometer (Thermo Fisher Scientific, San Diego, CA, U.S.A.). In the Global DNA Methylation LINE-1 kit, genomic DNA of interest is fragmented by enzymatic digestion and quantified using a NanoDrop ND-1000 again. After that, 320 ug fragmented DNA is hybridized with a biotinylated human LINE-1 consensus probe. Hybridized DNA is immobilized onto a streptavidin-coated plate, while unbounded DNA fragments are washed away. A 5-Methylcytosine antibody and a secondary antibody conjugated to horseradish peroxidase (HRP) are used for detection of methylated fragments. The colorimetric readout is easily quantified by spectrophotometry using a microplate reader at 450 nm and 655 nm. For quantitative analysis, the % 5-Methylcytosine associated with each sample can be calculated based on the CpG content: The 290 bp LINE-1 Msel fragments that hybridize to the LINE-1 probe contain 10 detectable CpG residues. The provided DNA standards are either 100% methylated or 0% methylated for all 10 CpGs. Therefore, the standard curve generated by mixing the DNA standards together quantitates methylation in the range of 0% to 100% 5- Methylcytosine for the CpG content of the samples. MasterPlex ReaderFit curve fitting software is available for data analysis.

### Statistical analysis

All data were presented as the mean ± SD from at least three independent experiments. FACS data sets were analyzed using FlowJo V10.6 software (FlowJo, LLC, Ashland, Oregon). A statistical analysis was performed using a paired t-test for CIK cells on day 14 in comparison to relative PBL cells. For multiple group comparisons one-way or two-way analysis of variance with the Dunnett *post hoc* test or Tukey *post hoc* test was performed via GraphPad Prism 9.0.0 software. P-values < 0.05 were considered significant differences and are marked: * < 0.05; ** < 0.01; *** < 0.001, ****p < 0.0001.

## Results

### CBD inhibits proliferation of A549 cells and induces p-γH2AX expression, apparently by involving TRPV2 channel regulation

**Figure 1:**
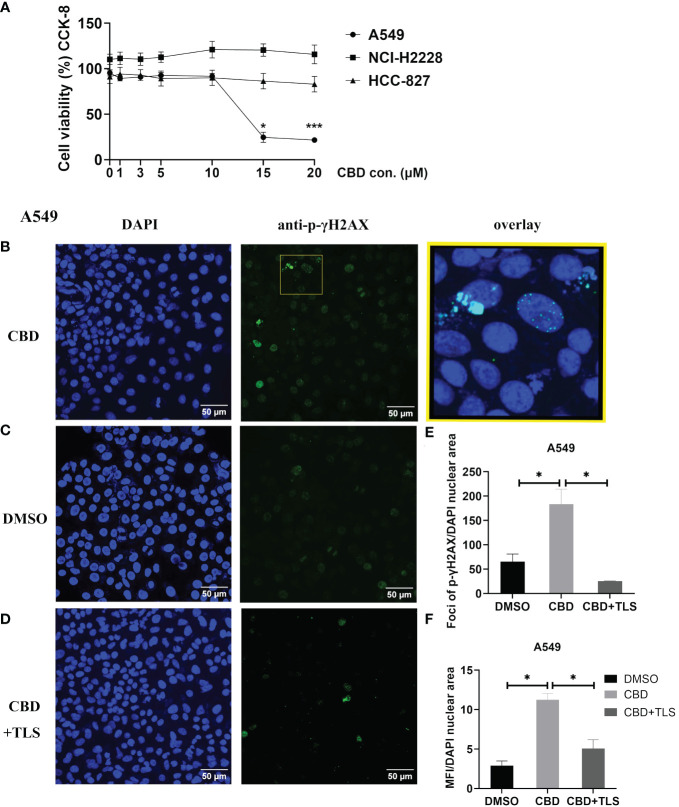
The cell viability of NSCLC cell lines and p-γH2AX expression after incubation with CBD. **(A)** The cell viability of NSCLC cell lines after incubation with CBD for 24 h was detected by CCK-8 assay. *p < 0.05, ***p < 0.001 vs. DMSO (0 µM) control. Data are shown as the mean ± SD, representative of five independent experiments. **(B)** Subcellular localization of p-γH2AX in A549 cells. A549 cells were incubated with CBD for 3 h with either DMSO, CBD or CBD and TLS, then fixed and immunolabeled. Nuclear fluorescent signals were captured by Visitron VisiScope Spinning Disk Confocal Microscopy and VisiVIEW^®^ Image software. The image analyses were facilitated by the FiJi ImageJ software. Left panel **(B–D)**, Nuclei of A549 cells were stained with blue signals for DAPI. Middle panel, A549 cells were immunolabeled of the anti-phospho-Histone H2AX (Ser139) antibody (clone JBW301) with green signals for Alexa Fluor 488. Upper right panel: a merged image of the DAPI and p-γH2AX signals in an enlarged yellow figure is presented. Scale bar = 50 µm. **(E, F)** Statistical analyses of Foci and MFI of p-γH2AX were carried out, respectively. *p < 0.05, CBD vs. DMSO control or CBD vs. CBD+TLS. All data are shown as the mean ± SD, representative of three independent experiments. Statistical analysis was performed using one-way ANOVA followed by Tukey’s multiple comparison test by GraphPad Prism software version 9.0.0.

It is generally believed that the ATM/ATR signaling pathway mediates G0–G1 arrest by regulating the expression of p53. Some investigations have reported that CBD-induced G1 arrest may be due to DNA damage via activation of ATM and downregulation of P53 expression in response to CBD (36, 37). Therefore, we chose A549 cells (KRAS mutation, P53 wide-type), NCI-H2228 (EML4-ALK variant 3, P53 Q331* mutation) and HCC-827 (EGFR exon19 deletion mutant, inactivation P53 mutation) with different genetic backgrounds. We investigated the dose-dependence of CBD in NSCLC cell lines and found that A549 appeared to be more sensitive compared to NCI-H2228 and HCC-827. A CBD dose-response curve for A549 is shown in **Figure 1A** (The cell viability at 15 µM CBD vs. DMSO 24.6 ± 5.6% vs. 95.37 ± 3.3%, P=0.0186; 20 µM CBD vs. DMSO 21.5 ± 2.5% vs. 95.37 ± 3.3%, P=0.0004). Since it cannot be excluded that certain CBD concentrations may cause DNA-related damage, we checked the early cellular response to the induction of DNA double-strand breaks by assaying phospho-γH2AX in NSCLC cells. We found that after 3 hours of CBD exposure, both foci and mean fluorescence intensity (MFI) of p-γH2AX were significantly increased compared with DMSO control in A549 cells (foci/total DAPI nuclear area: 183.6 ± 30.5 vs. 65.8 ± 15.4, P= 0.0483; MFI/total DAPI nuclear area: 11.3 ± 0.8 vs. 2.9 ± 0.6, P= 0.0130 (**Figures 1B–F**). Of interest, the enhancement of p-γH2AX expression was completely abolished when the TRPV2 channel antagonist tranilast (TLS) was co-administered with CBD at a dose of 10 µM (CBD vs. CBD+TLS, Foci/total DAPI nuclear area: 183.6 ± 30.5 vs. 25.6 ± 0.2, P= 0.0220; 11.3 ± 0.8 vs. 5.1 ± 1.2, P= 0.0300, respectively). However, no such changes were observed in NCI-H2228 and HCC-827 (**Supplementary Figures S1A, B**). The expression of p-γH2AX was not detectable in CIK cells after 3 h of incubation, neither with 10 µM CBD nor with DMSO control (**Supplementary Figure S1C**). Thus, it is suggested that CBD may actively increase the expression of p-γH2AX in NSCLC cell lines, particularly A549 (P53 wide-type genotype).

### CBD significantly suppressed the migration and invasion ability of NSCLC cells

**Figure 2:**
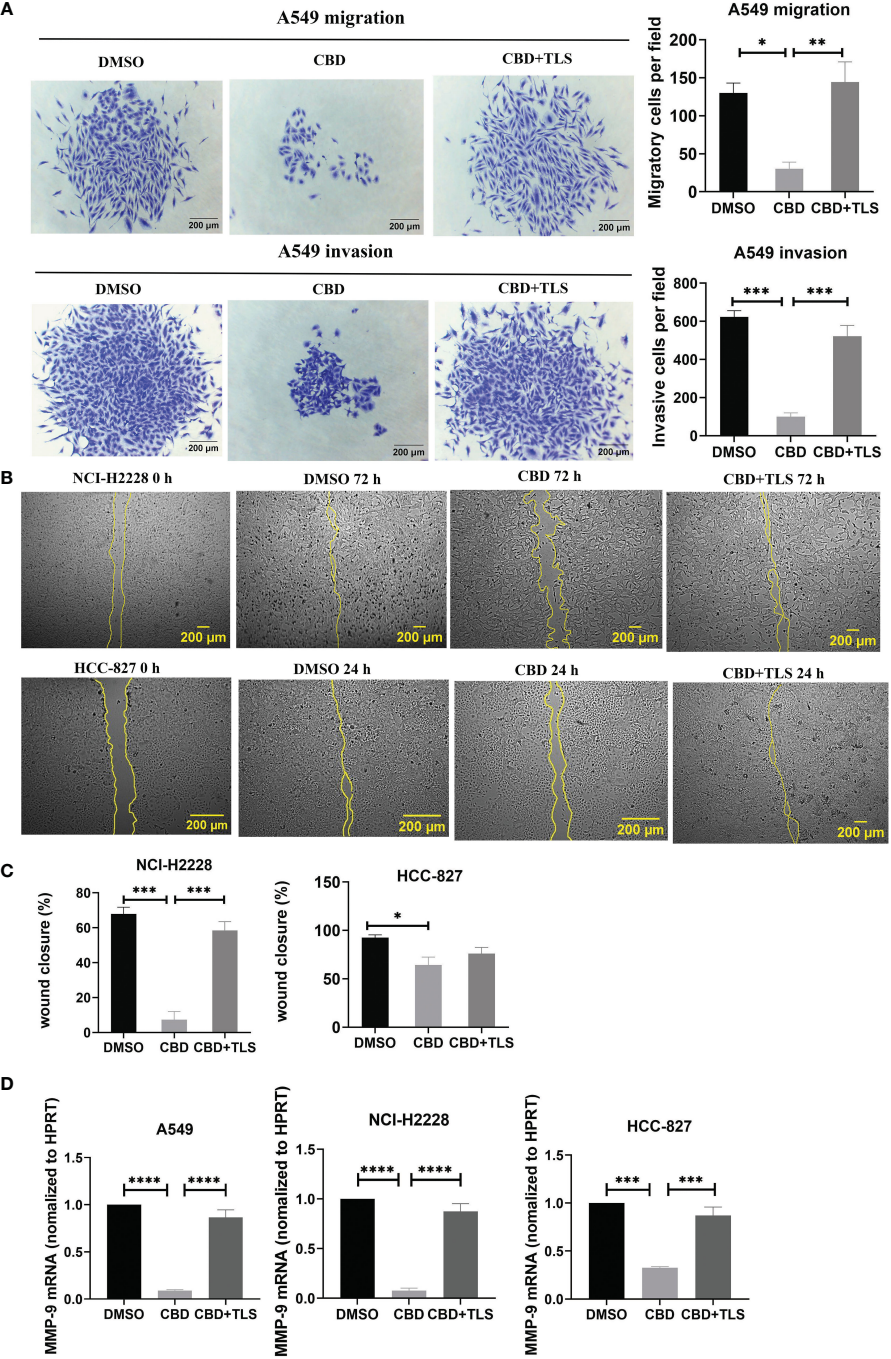
The migration and invasion of non-small cell line cancer cells after incubation with CBD. **(A)** A549 trans-well migration and invasion assays showed that A549 cells incubated with 10 µM CBD had lower migratory and invasive potentials than the control (DMSO control and 10 µM CBD with 10 µM TLS) for 72 h. **(B)** Effect of 10 µM CBD on NCI-H2228 or HCC-827 cell migration for 24-72 h in a scratch wound healing assay. The wound healing area was measured by the FiJi ImageJ software and the percentage of wound closure was calculated. **(C)** shows the statistical results. Data represented the mean ± SD of four independent experiments. **(D)** Quantitative analysis of mRNA levels of MMP-9 in tumor cells after incubation with CBD for 24h in free-serum RPMI 1640 medium. Phosphoribosyltransferase (HPRT) served as an internal standard. *p < 0.05, **p < 0.01, ***p < 0.001. Scale bar = 200 µm. All data are shown as the mean ± SD, representative of three independent experiments. Statistical analysis was performed using a one-way ANOVA followed by Tukey’s multiple comparison test by GraphPad Prism software version 9.0.0.

Like p-γH2AX expression, we subsequently investigated whether CBD may also influence the migration and invasion potential of NSCLC cell lines. The results revealed that CBD significantly suppressed the migration and invasion ability of A549 cells, and this inhibition was abolished by the TRPV2 antagonist tranilast (**Figure 2A**), (Migratory cells per field: CBD + A549 vs. DMSO control, P= 0.0177; CBD + A549 vs. CBD + TLS + A549, P= 0.0096. Invasive cells per field: CBD + A549 vs. DMSO control, P= 0.0002; CBD + A549 vs. CBD + TLS + A549, P= 0.0007, respectively). In contrast, we found that NCI-H2228 and HCC-827 cells were not capable of invading neither the membranes nor the Matrigel-coating membranes and they migrated fast and collectively. Considering that some cell types can migrate horizontally faster without invading a pore membrane (30), we therefore performed wound healing assays and observed significant migration ability for NCI-H2228 and HCC-827 (**Figures 2B, C**. CBD + NCI-H2228 vs. DMSO control, P= 0.0002; CBD + NCI-H2228 vs. CBD + TLS + NCI-H2228, P= 0.0009; CBD + HCC-827 vs. DMSO control, P= 0.0184; CBD + HCC-827 vs. CBD + TLS + HCC-827, P= 0.4017, respectively). In addition, we assessed the messenger RNA (mRNA) expression of matrix metalloproteinase-9 (MMP-9), which has been shown to represent the invasiveness of NSCLC (38). The level of mRNA of MMP-9 significantly was reduced after incubation with 10 µM CBD for 24 h in NSCLC cells (**Figure 2D**. CBD + A549 vs. DMSO control, P< 0.0001; CBD + A549 vs. CBD + TLS + A549, P< 0.0001; CBD+NCI-H2228 vs. DMSO control, P< 0.0001; CBD + NCI-H2228 vs. CBD + TLS + NCI-H2228, P< 0.0001; CBD+HCC-827 vs. DMSO control, P= 0.0002; CBD + HCC-827 vs. CBD + TLS + HCC-827, P= 0.0007, respectively). Collectively, CBD effectively inhibited the migration and invasion of NSCLC cell lines, presumably via the TRPV2 channel.

### TRPV2 highly expressed both in CIK cells and NSCLC cell lines

Immunocytochemistry revealed that most TRPV2 resides in an intracellular compartment. There is no TRPV2 present at the cell surface, both in CIK cells and NSCLC cell lines (**Supplementary Figure S2A**). Similarly, the intracellular percentage of FITC-TRPV2 was 98.92 ± 0.44% in CD3^+^CD56^+^ CIK cells, 99.95 ± 0.05% in CD3^+^CD4^+^ CIK cells, 99.82 ± 0.07% in CD3^+^CD8^+^ CIK cells detected by flow cytometric method. Moreover, the percentage of FITC-TRPV2 in A549 was 99.76 ± 0.23%, 99.9 ± 0.03% in NCI-H2228, and 91.95 ± 0.55% in HCC-827 (**Supplementary Figure S2B**).

### CBD promotes the cytotoxic activity of CIK cells in NSCLC cell lines, with A549 being the most sensitive concerning cytotoxicity

**Figure 3:**
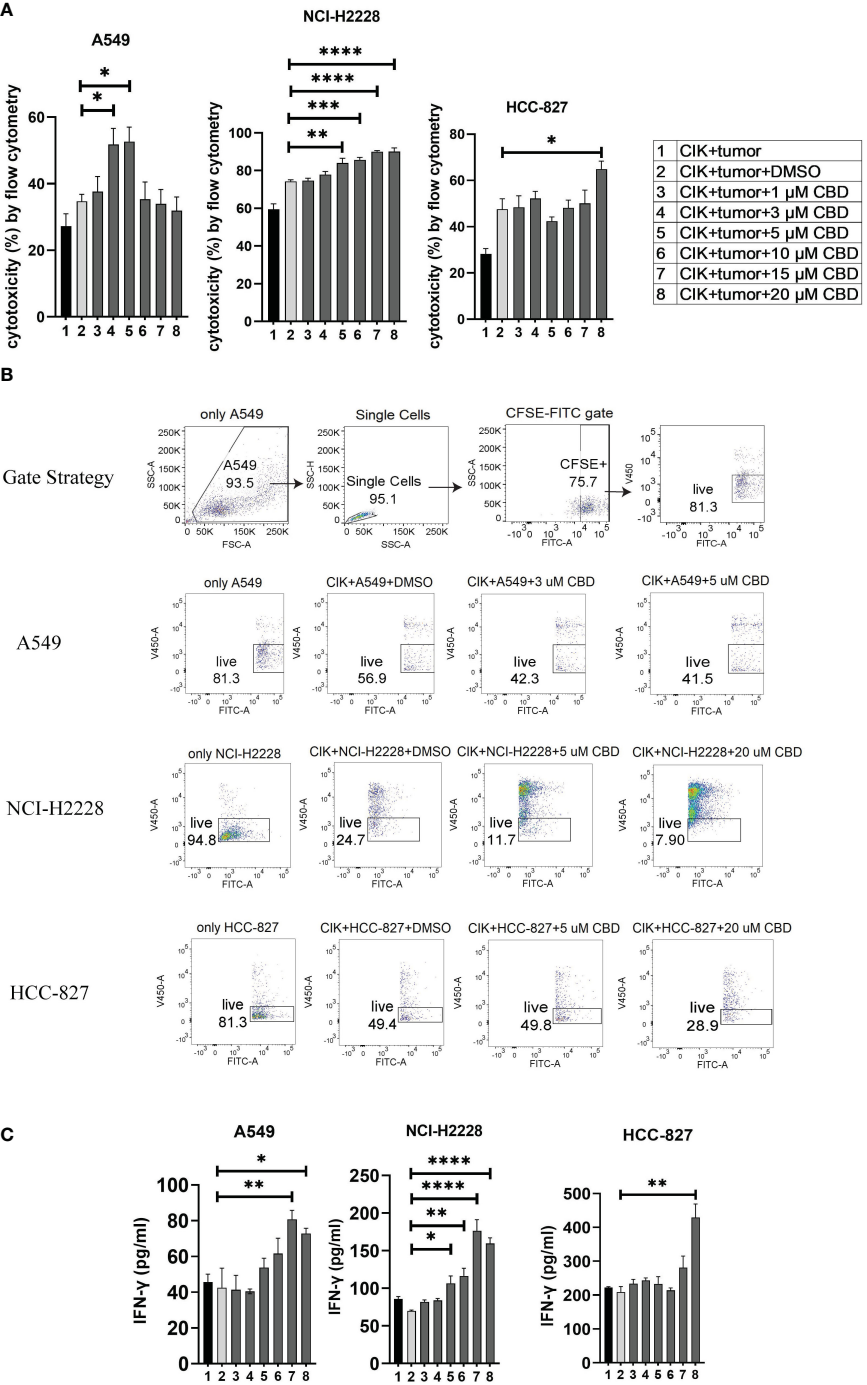
The cytotoxicity of CIK cells was measured using flow cytometry and IFN-γ secreted from CIK cells was detected by ELISA. **(A)** The cytotoxicity of CIK cells against A549, NCI-H2228 or HCC-827 with CBD treatment was detected by flow cytometry assessment after 24 h at different concentrations of CBD. **(B)** Representative flow cytometry plots illustrated the typical gating strategy used to identify viable tumor cells after treatment with a combination of CIK cells and CBD. **(C)** IFN-γ levels from CIK cells after treatment with a combination of CBD on NSCLC target cells detected by ELISA. E:T (effector-target) ratio =10:1. *p < 0.05, **p < 0.01, ***p < 0.001, ****p < 0.0001 vs. CIK combines DMSO targeting NSCLC cell control. Statistical analysis was performed using two-way ANOVA followed by Dunnett’s multiple comparison test by GraphPad Prism software version 9.0.0. Data are shown as the mean ± SD, representative of three independent experiments. CIK cells were derived from 3 donors.

To evaluate the effect of CBD on the cytotoxic activity of CIK cells, we prelabeled NSCLC cells with carboxyfluorescein diacetate succinimidyl ester (CFSE) and co-cultured them with CIK cells at an ET ratio of 10:1. It was found that the percentage of cytotoxicity of CIK cells against A549 was significantly increased after the combination of 3 µM CBD/5 µM CBD compared with DMSO control (51.8 ± 2.1% vs. 34.7 ± 3.6%, P< 0.05; 52.7 ± 4.4% vs. 34.7 ± 3.6%, P< 0.05, respectively). In the case of NCI-H2228, a significant increase was observed at 5 µM, 10 µM, 15 µM, and 20 µM CBD compared with the DMSO control after 24 hours of incubation (84.0 ± 2.1% vs. 74. 3 ± 0.9%, P= 0.0037; 85.7 ± 1.4% vs. 74.3 ± 0.9%, P= 0.0009; 90.0 ± 0.2% vs. 74.3 ± 0.9%, P < 0.0001; 90.0 ± 0.8% vs. 74.3 ± 0.9%, P< 0.0001, respectively). Whereas, 20 µM CBD appeared to be sufficient in case of HCC-827 (64.9 ± 3.5% vs. 47.6 ± 4.5%, P< 0.05) (**Figure 3A**). Representative flow cytometry plots illustrated the typical gating strategy used to identify the viable tumor cells after treatment with a combination of CIK cells and CBD (**Figure 3B**). Next, we examined the potential variations in IFN-γ cytokine release (due to cytotoxicity of CIK cells) and found that NCI-H2228 responded strongly for IFN-γ secretion when exposed to 5 µM, 10 µM, 15 µM, and 20 µM CBD concentrations (106. 7 ± 9.8 vs. 69.9 ± 1.7 pg/mL, P= 0.0251; 116.3 ± 10.3 vs. 69.9 ± 1.7 pg/mL, P= 0.0045; 176.3 ± 14.9 vs. 69.9 ± 1.7 pg/mL, P< 0.0001; 159.4 ± 7.6 vs. 69.9 ± 1.7 pg/mL, P < 0.0001). A549 and HCC-827 showed a significant increase in IFN-γ at a concentration of 15 µM/20 µM (**Figure 3C**). Thus, it can be concluded that CBD promotes the cytotoxic activity of CIK cells in all NSCLC cell lines, with IFN-γ secretion.

In addition, the cytotoxicity of single CBD on NSCLC cells or CIK cells were investigated by flow cytometry. In consistent with our results of proliferation, CBD significantly increased the cytotoxicity of A549 at 20 µM compared to DMSO (14.92 ± 5.23% vs. -3.69 ± 0.27%, P< 0.0001). However, there was no significant effect on NCI-H2228 at 20 µM CBD concentration compared to DMSO control (6.05 ± 5.14% vs. -0.25 ± 0.25%, P= 0.1046), HCC-827 (2.62% ± 1.57 vs. -0.6% ± 0.15, P= 0.7232) and CIK cells (0.62 ± 0.46% vs. -0.02 ± 0.04%, P= 0.1497) (**Supplementary Figure S3**).

### CBD in combination with CIK cells significantly alters the LINE-1 expression and methylation in NSCLC cell lines

**Figure 4:**
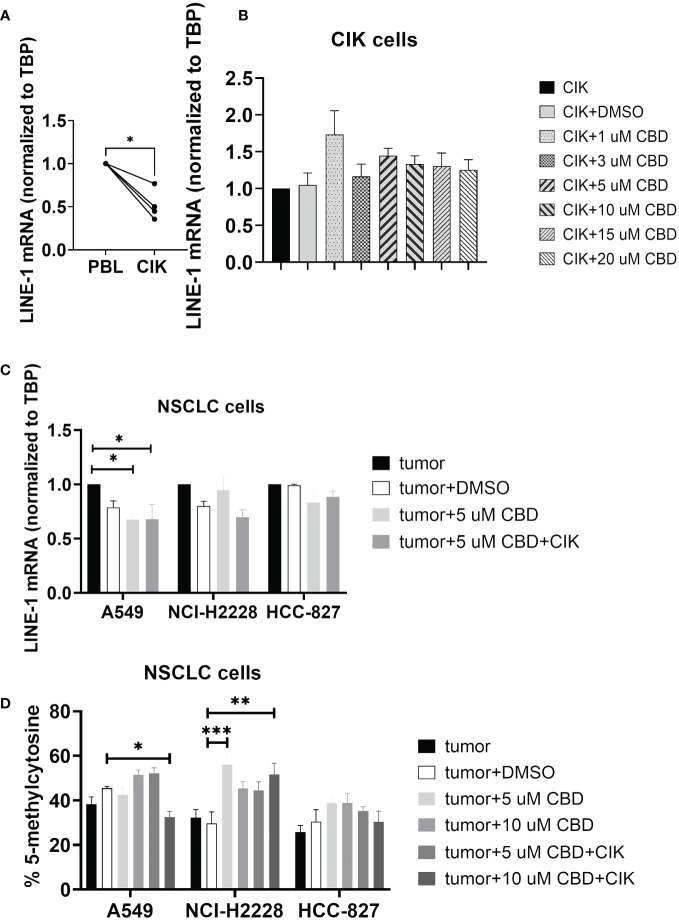
DNA genomic changes on CIK cells and NSCLC cells after incubation with CBD for 24 h. **(A)** LINE-1 mRNA expression in CIK cells on day 14 compared to PBL **(B)** LINE-1 mRNA expression in CIK cells after incubation with CBD for 24 h **(C)** LINE-1 mRNA expression in NSCLC cells after incubation with CBD or CBD with CIK cells. E:T ratio =0.1:1 **(D)** %5-mC associated with the detectable CpG residues in NSCLC cells after incubation with CBD or CBD with CIK cells. E:T ratio =0.1:1. All data are shown as the mean ± SD, representative of four independent experiments. Statistical analysis was performed using a paired *t test* for CIK cells on day 14 in comparison to relative PBL cells. A Two-way ANOVA followed by Dunnett’s multiple comparisons test was performed on another data using GraphPad Prism software version 9.0.0. *p < 0.05, **p < 0.01, ***p < 0.001.CIK cells were derived from 4 donors.

Limited evidence suggests that CBD may modulate epigenetic-related changes (39–41). While the main focus of studies remains on genomic DNA methylation, any alterations in the repetitive genome (e.g. LINE-1) have never been investigated. We therefore focused on the effect of CBD on the CIK cells alone and in combination with NSCLC cell lines, especially for global LINE-1 expression and methylation. We found that the level of mRNA of LINE-1 was significantly reduced on CIK day 14 compared to PBL control (P= 0.0122) (**Figure 4A**). CBD alone has no effect on the expression of LINE-1 in CIK cells (**Figure 4B**), however in combination it can significantly reduce the LINE-1 expression in A549 cells at an E:T ratio of 0.1:1 (**Figure 4C**, 5 µM CBD + A549 vs. tumor alone, P= 0.0436; 5 µM CBD + A549 + CIK vs. tumor alone, P= 0.0436, respectively). Similarly, 10 µM CBD with CIK cells significantly decreased the level of global LINE-1 DNA methylation in A549 (**Figure 4D**, %5-mC associated with detectable CpG residues: 10 µM CBD + A549 + CIK vs. DMSO + A549, 45.5 ± 1.1% vs. 32.6 ± 3.5%, P= 0.0388). Significant alterations were also observed in NCI-H2228 compared to DMSO control (5 µM CBD + NCI-2228 vs DMSO, 56.1 ± 8.6% vs. 29.7 ± 7.2%, P= 0.0002; 10 µM CBD + NCI-H2228 + CIK vs DMSO, 51.6 ± 7.2% vs 29.7 ± 7.2%, P= 0.0026, respectively). However, no change was noticed in HCC-827 cells.

### Mechanistically, CBD affects NKT subpopulations of CIK cells and may modulate TRPV2 channel and the p-ERK1/2 pathway

**Figure 5:**
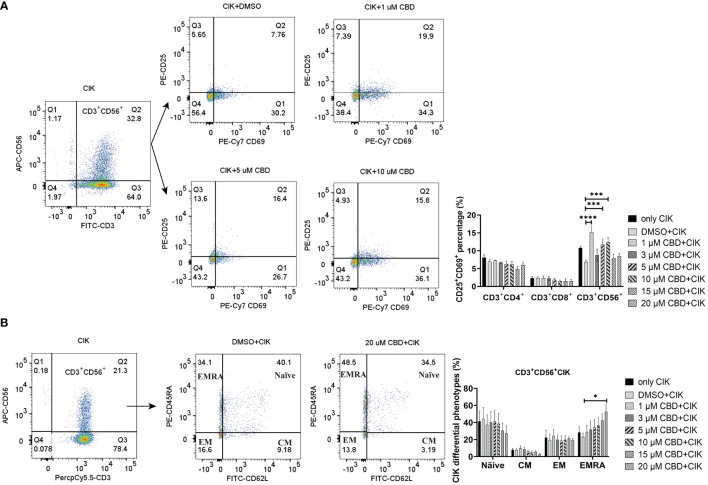
The phenotypes of CIK cells were measured using flow cytometry after incubation with CBD for 24 h. **(A)** The surface expression of CD25 and CD69 on CIK cells after 24 h of CBD treatment was detected by flow cytometry assessment at different concentrations of CBD. **(B)** The surface expression of CD45RA and CD62L on CIK cells after 24 h of CBD treatment was detected by flow cytometry assessment at different concentrations of CBD. Naïve: CD45RA^+^CD62L^+^ T cells, CM: central memory T cells (CD45RA^−^CD62L^+^), EM: effector memory T cells (CD45RA^−^CD62L^−^), EMRA: terminal effector memory T cells (CD45RA^+^CD62L^−^). *p < 0.05, ***p < 0.001, ****p < 0.0001 vs. CIK combined with DMSO control. Statistical analysis was performed using two-way ANOVA followed by Dunnett’s multiple comparison test by GraphPad Prism software version 9.0.0. CIK cells were derived from 4 donors.

Given that CIK cells are a heterogeneous cell population, among which NKT cells constitute a significant proportion. We therefore investigated the effect of CBD on the CIK cell populations, particularly the receptors associated with NKT activation [CD25 and CD69 (42)] present on the surface of CIK cells. To mention, CD69 is a useful marker for the cytotoxic activity of NK cells, whereas CD25 expression serves as an indicative of the proliferative potential. We found that the expression of CD25^+^CD69^+^ on the CD3^+^CD56^+^ CIK cells increased significantly when CIK cells were incubated with 1 µM, 5 µM, and 10 µM CBD for 24 h (**Figure 5A**). In addition, the percentage of CD25^+^CD69^+^ NKT cells increased after 24 h activation by 1 µM CBD compared with the DMSO control (15.2 ± 3.2% vs. 6.9 ± 1.0%, P< 0.0001). The isotype PE-Cy7 IgG1k was a PE-Cy7-CD69 control and the isotype PE-IgG1k was a PE-CD25 control (**Supplementary Figure S4B**). Also, CD45RA and CD62L expression patterns were used to identify naïve T cells (CD45RA^+^CD62L^+^), central memory T cells (TCM; CD45RA^-^CD62L^+^), effector memory T cells (TEM; CD45RA-CD62L-), and terminal effector memory T cells (TEMRA; CD45RA^+^CD62L^-^). We found significantly high percentage of CD45RA^+^CD62L^-^ TEMRA terminal effector memory NKT subset of CIK cells at 20 µM CBD for 24 h compared with DMSO control (52.7 ± 16.5% vs. 23.1 ± 9.1%, P= 0.0473, **Figure 5B**). In other subsets of CIK cells, no significant differences were seen (**Supplementary Figure S4A**). Thus, suggesting that CBD may contribute to the cytotoxic activation phase of CD3^+^CD56^+^CIK cells.

**Figure 6:**
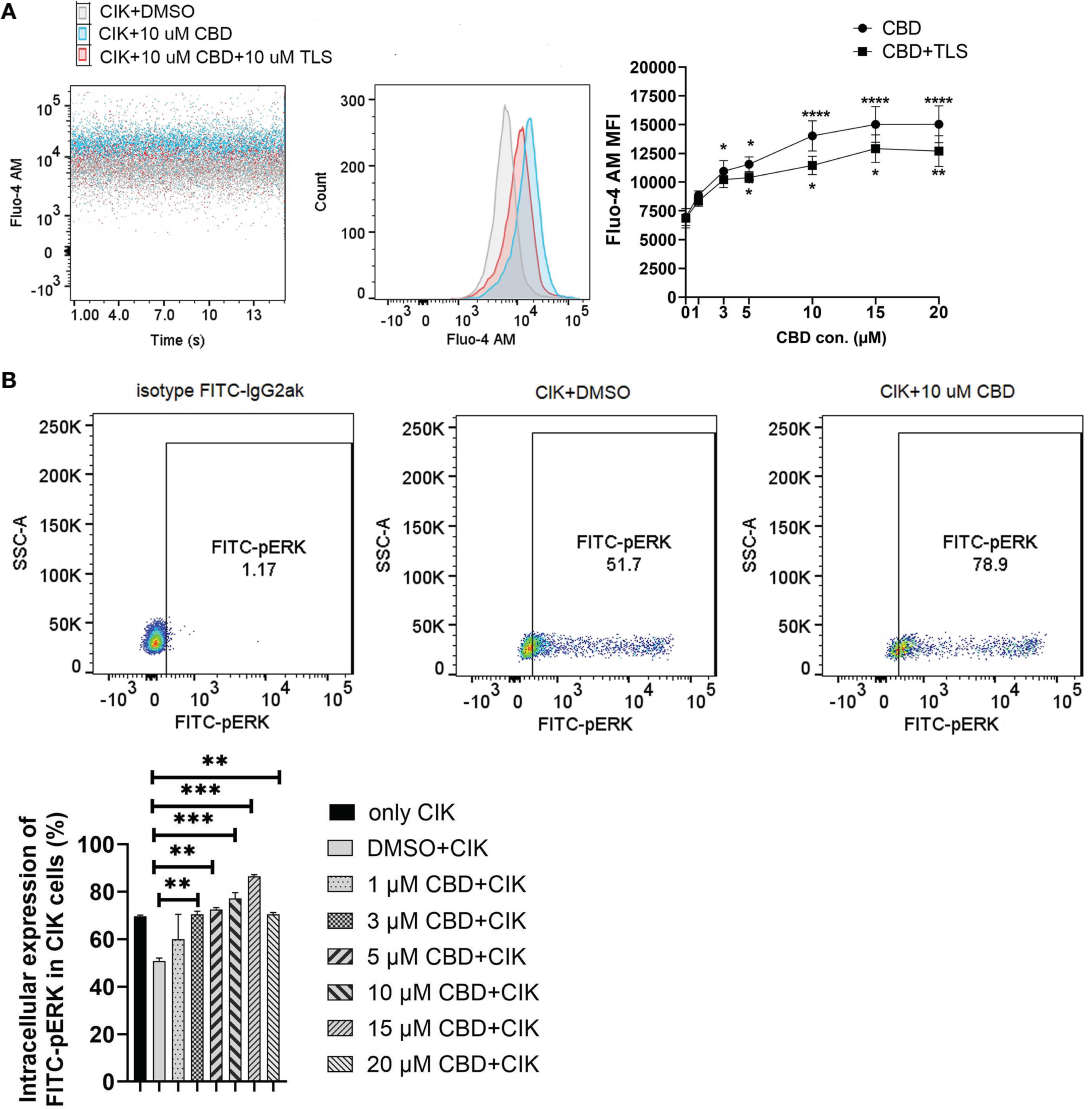
Characterization of intrinsic alternation in the CIK cells in the CBD-inducing culture by flow cytometric analysis. **(A)** CIK cells were exposed to either CBD or CBD combined with the TRPV2 channel antagonist tranilast at 37°C for 1 minute. Dead cells were gated and excluded by Hoechst 33258 for Fluo-4 AM expression. **(B)** The phosphorylation levels of Erk1/2 in CIK cells were determined by flow cytometry. Dead cells were gated and excluded by Zombie Aqua™ dye for intracellular expression. The rabbit of isotype controls is indicated. The percentage of FITC anti-ERK1/2 Phospho (Thr202/Tyr204) was analyzed using the Flowjo V10 software. *p < 0.05, **p < 0.01, ***p < 0.001, ****p < 0.0001 vs. CIK combined with DMSO control. All data are shown as the mean ± SD, representative of four independent experiments. Statistical analysis was performed using a two-way ANOVA followed by Dunnett’s multiple comparisons test by GraphPad Prism software version 9.0.0. CIK cells were derived from 4 donors.

Considering that CBD can also act via transient receptor potential vanilloid-2 (TRPV2), we therefore evaluated the intracellular calcium level in CIK cells by treating them using variable concentrations of CBD and the specific TRPV2 channel antagonist tranilast (TLS). Interestingly, we observed that CBD as a TRPV2 agonist significantly increased intracellular Ca^2+^ levels in CIK cells at 3 µM, 5 µM, 10 µM, 15 µM, and 20 µM CBD after 1 minute (mean fluorescence intensity MFI 10943.63 ± 921.61 vs. 6966.25 ± 739. 06, P= 0.0373; 11545.75 ± 650.99 vs. 6966.25 ± 739.06, P= 0.0126; 14021 ± 1309.35 vs. 6966.25 ± 739.06, P < 0.0001; 15022.88 ± 1547.10 vs. 6966.25 ± 739.06, P< 0.0001; 15035.88 ± 1604.35 vs. 6966.25 ± 739.06, P< 0.0001, respectively). On the contrary, 10 µM tranilast significantly affected the intracellular Ca^2+^ levels with a mean of 9.96% (5 µM CBD+10 µM TLS vs. 5 µM CBD, P= 0.0298), 18.35% (10 µM CBD+10 µM TLS vs. 10 µM CBD, P= 0.0342), 14.07% (15 µM CBD+15 µM TLS vs. 15 µM CBD, P= 0.0110), and 15.54% (15 µM CBD+15 µM TLS vs. 10 µM CBD, P= 0.0342), 14.07% (15 µM CBD+15 µM TLS vs. 15 µM CBD, P= 0.0110), and 15.54% (20 µM CBD+20 µM TLS vs. 20 µM CBD, P= 0.0095) (**Figure 6A**). This outcome suggests that CBD may promote intracellular calcium through the TRPV2 channel. Continuing the analysis, we also found a significant increase in the percentage of intracellular expression of FITC anti-ERK1/2 phospho (Thr202/Tyr204) in CIK cells after incubation with CBD at concentrations of 3, 5, 10, 15, 20 µM for 15 min compared with DMSO control (**Figure 6B**). Meanwhile, we investigated the effect of FR180204 (a selective inhibitor of ERK) on the cytotoxicity of CBD-treated CIK cells against NSCLC cells. FR180204 abolished CBD-induced cytotoxicity of CIK cells against A549 (CBD group: 3 µM CBD vs. DMSO, P= 0.0002, 5 µM CBD vs. DMSO, P< 0.0001; FR18028+CBD group: 10 µM FR18028 + 3 µM CBD vs. DMSO, P= 0.9811, 10 µM FR18028 + 5 µM CBD vs. DMSO, P= 0.9825, respectively). Similarly, Pretreated-FR180204 in CIK cells reduced CBD-induced cytotoxicity of CIK cells against NCI-H2228 (CBD group: 5 µM CBD vs. DMSO, P= 0.0002; 10 µM CBD vs. DMSO, P= 0.0002; 15 µM CBD vs. DMSO, P= 0.0002; 20 µM CBD vs. DMSO, P< 0.0001; FR18028+CBD group: 10 µM FR18024 + 5 µM CBD vs. DMSO, P= 0.4064; 10 µM FR18024 + 10 µM CBD vs. DMSO, P= 0.5480; 10 µM FR18024 + 15 µM CBD vs. DMSO, P= 0.4192; 10 µM FR18024 + 20 µM CBD vs. DMSO, P= 0.1627, respectively) and HCC-827 (CBD group: 20 µM CBD vs. DMSO, P< 0.0001; FR18028+CBD group: 10 µM FR18028 + 20 µM CBD vs. DMSO control, P= 0.2535, respectively) (**Supplementary Figure S5**). Overall, these results suggest that CIK cells are activated by CBD through the TRPV2 channel and CBD-induced cytotoxicity is regulated by the ERK pathway.

## Discussion

There have been several ongoing efforts to understand the molecular function of cannabidiol (CBD) in cancer (43), primarily as a potential anti-cancer agent with relevance to multiple clinical therapies. Herein, we sought to understand the functional aspect of CBD in the preclinical lung cancer model and its compatibility with cytokine-induced killer (CIK) cell immunotherapy (44), which is now approved in several countries, including Germany. We primarily addressed, 1) whether CBD has any particular effects on NSCLC cell lines with diverse genetic backgrounds, 2) whether CBD can exert any influence on CIK cells aiming to enhance their therapeutic potential/function in NSCLC cells, 3). If CBD combined with CIK cells can affect the expression and methylation of LINE-1 repetitive sequences in NSCLC cells.

To determine this, we first investigated whether CBD has any particular effects on NSCLC cell lines with diverse genetic backgrounds such as A549 cells (KRAS mutation, P53 wide-type), NCI-H2228 (EML4-ALK variant 3, P53 ^Q331*^ mutation) and HCC-827 (EGFR exon19 deletion mutant, inactivation P53 mutation). A study concluded that CBD treatment regulates several types of cell death via upregulating apoptosis-related proteins, such as p53 in A549 (45). Similarly, our analysis revealed that A549 cells were more sensitive to CBD treatment compared to other cell types, presumably attributable to their p53 wild-type state. These findings are consistent with the notion that marijuana smoke condensates (MSCs) induce DNA/chromosome damage and apoptosis in human lung cancer cells, and the apoptotic responses induced by MSCs appeared to be higher in p53-WT H460 cells than in p53-null H1299 cells (46). The expression of p-γH2AX increased significantly in A549 in the presence of CBD, suggesting that CBD may promote DNA DSBs, p53 may also partially contribute here. Similar to p-γH2AX expression, we subsequently investigated whether CBD may also influence the migration and invasion potential of NSCLC cell lines. Using Matrigel invasion assays and wound healing assays we found cannabidiol-driven impaired migration in NSCLC cells that was reversed by antagonists to transient receptor potential vanilloid 2 (TRPV2). The decrease in invasion by cannabidiol appeared concomitantly with the downregulation of matrix metalloproteinases-9 (MMP-9). Interestingly, there is several evidence supporting the role of CBD in TRPV1-elicited p42/44 MAPK activation and downstream TIMP-1-dependent inhibition of invasion (47, 48). TIMP-1, as a strong inhibitor of MMP-9 activation and activity, keeps the intracellular and nuclear MMP-9 activation status under surveillance in a healthy retina (49). Higher expression of MMP-9 in cancerous tissue in NSCLC patients compared to non-cancerous tissue, and unchanged expression of TIMP-1, suggest imbalanced regulation of MMP-9/TIMP-1 (50). In our study, TRPV2-dependent MMP-9 reduction also exhibits a pivotal role in the anti-invasive action of CBD. Collectively, CBD might modulate the TIMP1/MMP9 gene expression axis to impair the metastasis of NSCLC cells.

Next, we asked, whether CBD can exert any influence on CIK cells aiming to enhance their therapeutic potential function in NSCLC cells. We confirmed that CBD promotes the cytotoxic activity of CIK cells in NSCLC cell lines, with A549 being the most sensitive concerning cytotoxicity. To subvert the exact cell population within CIK cells as a target of CBD, we performed phenotype analysis and found that the expression of CD25^+^CD69^+^ NKT on CD3^+^CD56^+^ CIK cells increased significantly when CIK cells were incubated with CBD. To mention, our findings underscore the impact of CBD on the differentiation of terminal effector memory (EMRA) (CD45RA^+^CD62L^−^) NKT CIK cells rather than CD4^+^ or CD8^+^ CIK cells, revealing that the desired terminal effector memory NKT response might contribute to the cytotoxicity of CIK cells in the presence of CBD. Since it has been suggested that CBD interacts with TRPV2 through a hydrophobic pocket (4), we therefore examined intracellular calcium levels in CIK cells by treating them with CBD and the TRPV2 channel antagonist tranilast (TLS). Interestingly, we found that CBD as a TRPV2 agonist significantly increased intracellular Ca^2+^ levels in CIK cells and the p-ERK1/2 pathway. To our knowledge, this is the first study to provide evidence of activation of CIK cells by CBD that is related to Ca^2+^ influx and modulated by TRPV2 channel activation.

Five distinct types of ion channels – Kv1.3, KCa3.1, Orai1+ stromal interacting molecule 1 (STIM1) [Ca^2+^-release activating Ca^2+^ (CRAC) channel], TRPM7, and Cl(swell) – comprise a network that performs functions vital for ongoing cellular homeostasis and for T-cell activation, offering potential targets for immunomodulation (51). Orai1 and STIM1 have been found to move to the immunological synapse and are up-regulated during T cell activation (52). More importantly, TRPV2 has been found to cluster at the immunological synapse following contact with antigen-presenting cells, together with Kv1.3, KCa3.1, STIM1, and Orai1 channels (53). In addition, expression of TRPV can be upregulated in T cells during concanavalin A-driven mitogenic and anti-CD3/CD28 stimulated TCR activation of T cells. By specific blocking of TRPV1 and TRPV4 channels, it was observed that these TRPV inhibitors may regulate mitogenic and TCR-mediated T cell activation and effector cytokine(s) production by suppressing tumour necrosis factor, interleukin-2 and interferon-γ release (54).

In our study, cannabidiol regulates ion channel TRPV2 of CIK cells by increasing intracellular Ca^2+^ in T-cell activation at an early stage, which might activate tyrosine kinase Zap70 (TCR pathway) or move to immunological synapses like Orail and STIMI. ERK inhibitor FR180204 blocked CBD enhancement of CIK cell cytotoxicity, suggesting that it is mediated by ERK signalling downstream of the Ca^2+^ influx. After 24 h of incubation, CBD regulates CIK differentiation and effector cytokine IFN-γ from CIK cells. How TRPV2 interacts with TCR needs further exploration by application of Hydrogen-Deuterium Exchange Mass Spectrometry (HDX-MS) for analyzing structural features and dynamic properties of proteins (55).

It is worth mentioning that the therapeutic potential of CBD in combination with epigenetic and classical chemotherapy drugs is of importance (40), but the biological (unfavorable) effects of CBD itself on the epigenetic system cannot be excluded. There have been studies focusing on CBD-induced abnormal changes in DNA methylation (39, 56, 57), however, to our knowledge, the effect of CBD on long interspersed element 1 (LINE-1 or L1) remains elusive. In cancer cells, long interspersed element-1 (LINE-1 or L1) is a repetitive DNA retrotransposon that duplicates via a copy-and-paste genetic mechanism. With LINE-1 comprising a substantial portion (approximately 17%) of the human genome, its methylation often correlates with global genomic methylation (58). As we mentioned before, LINE-1 promotes tumorigenicity and exacerbates tumor progression in NSCLC cell lines and mice model (22). A recent study revealed that LINE1 elements were specifically expressed in the nuclei of näive CD4^+^ T cells (RNA-FISH and RT–qPCR). LINE-1 expression in Th1 cells was rapidly downregulated upon activation and remained at low levels during differentiation (23). Our results in **Figure 5A** are consistent with this study. When CIK cells were activated and matured at day 14, LINE-1 mRNA significantly decreased. It seems like higher LINE-1 expression demonstrates the resting stage of T cells. In the current study, we observed that the combination of CIK cells and CBD suppressed LINE-1 mRNA and also decreased LINE-1 DNA methylation levels in A549 cells. Redi E, et al. discovered aberrant DNA methylation patterns in ALK^+^ tumor cells, overlapping with regulatory regions, plus a high degree of epigenetic heterogeneity between individual tumors. This discrepancy might be associated with an increase of DNA methylation of LINE-1 in NCI-H2228 in the presence of CBD (59). Importantly, CBD alone had no effect on mRNA LINE-1 expression or p-rH2AX expression in CIK cells, suggesting CBD does not lead to genome instability or DNA damage in CIK cells.

This study has limitations. Firstly, we did not investigate the effects of CBD distinctly in squamous cell carcinomas (SCC) subgroup and cancer stem cells. Hamad H. et al. found that CBD decreased viability and induced cell death in NSCLC (A549, H1299), SCLC (H69) and decreased the cancer stem cell spheres of both NSCLC and SCLC (10). Ramer R. et al. reported that treatment with 5 mg/kg CBD inhibits lung cancer cell invasion and metastasis via intercellular adhesion molecule-1 in athymic nude mice xenografted with A549 cells (60) while there was no evidence for *in vivo* investigations of the antimetastatic effect of CBD in SCLC. However, there is evidence that prognostic value of MMP-9 may be of more importance in adenocarcinoma than in SCC. In addition, MMP-9 was significantly associated with both disease-free survival and OS in the adenocarcinoma group (61). Secondly, the suppressive effect of combinations of CBD with THC or tyrosine kinase inhibitors or immune checkpoint inhibitors on lung cancer cells might also be examined in the future (62). Thirdly, in athymic nude mice xenografted with A549 cells, cannabidiol was found to reduce tumor volume significantly (63) and revealed a significand inhibition of A549 lung metastasis compared with vehicle-treated animals (47, 60). The effectiveness of a combination of CIK and CBD in the murine model will be further investigated.

## Conclusions

In conclusion, CBD holds a great potential for treating NSCLC with CIK cell immunotherapy and its complete success requires careful consideration of the patients’ genetic backgrounds. Cell lines with KRAS mutation (A549 cells) and EML4-ALK rearrangement (NCI-H2228) appear to be highly responsive in this combinatorial setup. Beyond that, CBD affects NKT subpopulations of CIK cells and may modulate the TRPV2 channel and the p-ERK1/2 pathway. However, the biosafety of a combination of CIK cells and CBD requires further validation in animal models.

## Data availability statement

The raw data supporting the conclusions of this article will be made available by the authors, without undue reservation.

## Author contributions

YL: Formal analysis, Investigation, Conceptualization, Data curation, Methodology, Resources, Software, Validation, Visualization, Writing – original draft, Writing – review & editing. AS: Conceptualization, Data curation, Methodology, Writing – review & editing. MH: Conceptualization, Methodology, Writing – review & editing. DS: Conceptualization, Writing – review & editing. ME: Writing – review & editing. HW: Methodology, Supervision, Writing – review & editing. IS-W: Conceptualization, Funding acquisition, Project administration, Supervision, Writing – review & editing.

## Supplementary material

Supplementary Figure 1 The effect of CBD on foci and MFI of p-γH2AX in NCI-H2228, HCC-827 and CIK cells. **(A)** NCI-H2228 cells or **(B)** HCC-827 cells or **(C)** CIK cells were incubated with CBD for 3 h with either DMSO, CBD or CBD combined with tranilast (TLS), then fixed and immunolabeled. Nuclear fluorescent signals were captured by Visitron VisiScope Spinning Disk Confocal Microscopy and VisiVIEW® Image software. The image analyses were facilitated by the FiJi ImageJ software. The nuclei of cancer cells were stained with blue signals for DAPI and phospho-Histone H2AX (Ser139) antibody (clone JBW301) with green signals for Alexa Fluor 488. Scale bar = 50 µm. All data are shown as the mean ± SD, representative of three independent experiments. Statistical analyses of Foci and MFI of p-γH2AX were carried out using one-way ANOVA followed by Tukey’s multiple comparison test by GraphPad Prism software version 9.0.0.

Supplementary Figure 2 Subcellular localization of TRPV2 in NSCLC cell lines and CIK cells detected by IHC and flow cytometry. **(A)** Cells were fixed and stained with NorthernLights™ NL557 with orange signals for TRPV2 and blue signals for nuclear DAPI. Fluorescent signals were captured by Visitron VisiScope Spinning Disk Confocal Microscopy and VisiVIEW® Image software. Scale bar = 50 µm. **(B)** CIK cells or NSCLC cells were stained with a Fixable Viability Zombie Aqua™ Dye exclude dead cells. After that, CIK cells were stained by APC anti-human CD3 antibody (clone OKT3), Brilliant Violet 421 anti-human CD8 antibody (clone RPA-T8), APC/cyanine7 anti-human CD4 antibody (clone RPA-T4), PE anti-human CD56 antibody (clone 5. 1 H11) at 4°C for 20 min. Afterwards, the cells were fixed and permeabilized using fixation buffer and intracellular staining permeabilization wash buffer. The blocking step was carried out in permeabilization wash buffer with 2% normal goat serum for 15 min. Cells were incubated with anti-TRPV2 antibody (1:100 dilution) at 4°C for 30 min. Subsequently, cells were washed and incubated with Alexa Fluor™ 488-labeled goat anti-rabbit IgG (H+L) (1:250 dilution) for 30 min.

Supplementary Figure 3 The cytotoxicity of CBD on single NSCLC cells or single CIK cells. **(A)** The cytotoxicity of CBD on NSCLC cells and the cytotoxicity of CBD on CIK cells. 5x10^4^ CFSE-labeled NSCLC cells were incubated with CBD for 24 h. The cytotoxicity of CBD on CIK cells. 5x10^5^ CIK cells were incubated with CBD for 24 h. **(B)** Representative flow cytometry plots illustrated the typical gating strategy used to identify viable CIK cells after treatment with CBD. **(C)** Representative flow cytometry plots illustrated to identify viable CFSE-labeled NSCLC cells after treatment with CBD. *p < 0.05, **p < 0.01, ***p < 0.001, ****p < 0.0001 vs. Cells combined with DMSO were designed as a control. Statistical analysis was performed using a two-way ANOVA followed by Dunnett’s multiple comparison test by GraphPad Prism software version 9.0.0. Data are shown as the mean ± SD, representative of four independent experiments. CIK cells were derived from 4 donors.

Supplementary Figure 4 **(A)** The surface expression of CD45RA and CD62L on CD3^+^CD8^+^CIK or CD3^+^CD4^+^ CIK cells after 24 h CBD treatment was detected by flow cytometry assessment. Naïve: CD45RA^+^CD62L^+^ T cells, CM: central memory T cells (CD45RA^−^CD62L^+^), EM: effector memory T cells (CD45RA^−^CD62L^−^), EMRA: terminal effector memory T cells (CD45RA^+^CD62L^−^). Statistical analysis was performed using a two-way ANOVA followed by Dunnett’s multiple comparison test by GraphPad Prism software version 9.0.0. CIK cells were derived from 4 donors. **(B)** PE-CD25 and PE-Cy7-CD69 gate strategies. The isotype PE-Cy7 IgG1k was a PE-Cy7-CD69 control and the isotype PE-IgG1k was a PE-CD25 control.

Supplementary Figure 5 The effects of ERK inhibitor FR180204 on the cytotoxicity of CBD-treated CIK cells against NSCLC cells. CIK cells were pretreated with 10 µM FR18024 for 2 h then cocultured with CFSE-labeled A549 **(A)**, NCI-H2228 **(B)** and HCC-827 **(C)** at different CBD concentrations. E:T (effector-target) ratio =10:1. *p < 0.05, **p < 0.01, ***p < 0.001, ****p < 0.0001 vs. CIK combined DMSO targeting NSCLC cells was designed as a control. Statistical analysis was performed using a two-way ANOVA followed by Dunnett’s multiple comparison test by GraphPad Prism software version 9.0.0. Data are shown as the mean ± SD, representative of three independent experiments. CIK cells were derived from 3 donors.
