# Supplementary figures and images for: Discovering single cannabidiol or synergistic antitumor effects of cannabidiol and cytokine-induced killer cells on non-small cell lung cancer cells
Source: Front Immunol. 2024 Mar 14;15:1268652. doi: 10.3389/fimmu.2024.1268652 (PMC10979545; doi:10.3389/fimmu.2024.1268652)

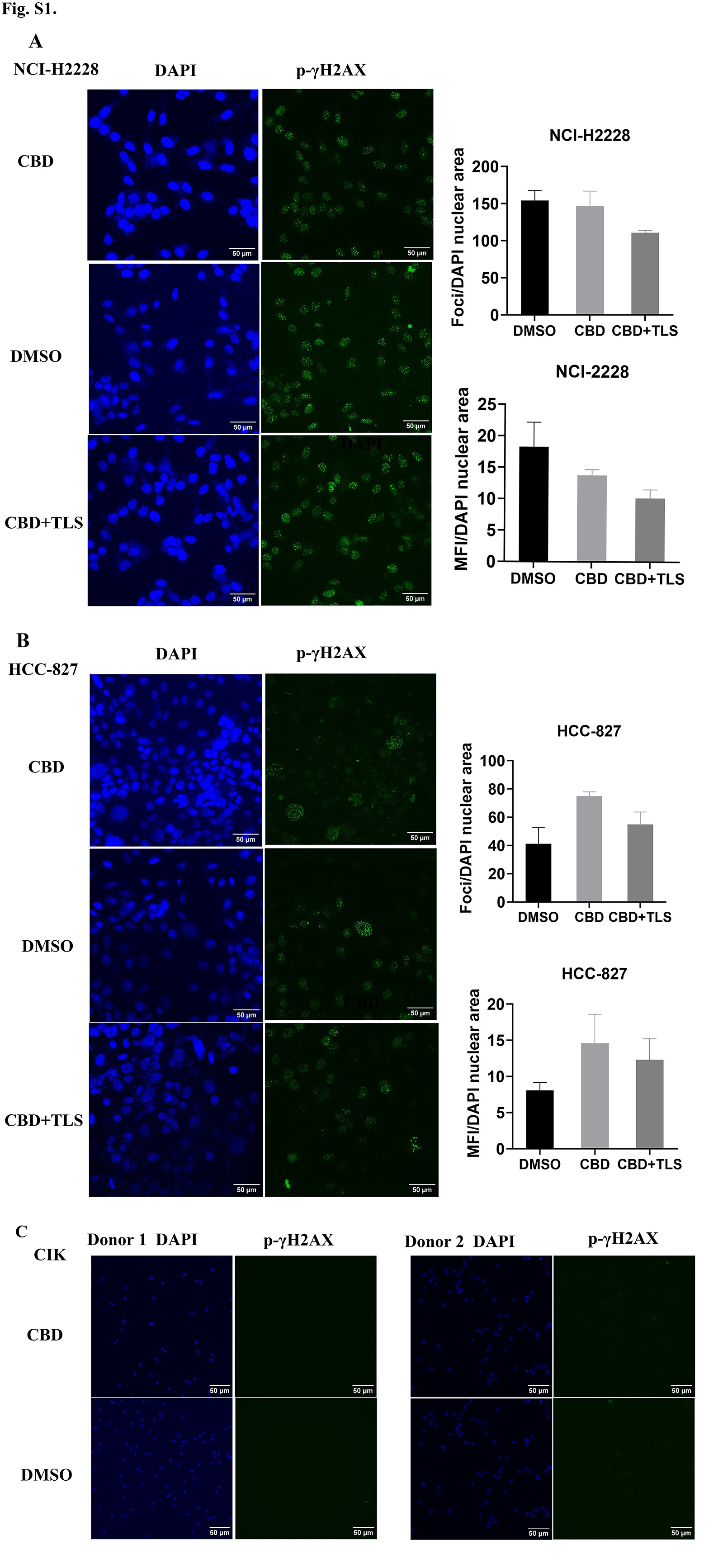

Supplement: Supplementary Figure 1 — The effect of CBD on foci and MFI of p-γH2AX in NCI-H2228, HCC-827 and CIK cells. (A) NCI-H2228 cells or (B) HCC-827 cells or (C) CIK cells were incubated with CBD for 3 h with either DMSO, CBD or CBD combined with tranilast (TLS), then fixed and immunolabeled. Nuclear fluorescent signals were captured by Visitron VisiScope Spinning Disk Confocal Microscopy and VisiVIEW® Image software. The image analyses were facilitated by the FiJi ImageJ software. The nuclei of cancer cells were stained with blue signals for DAPI and phospho-Histone H2AX (Ser139) antibody (clone JBW301) with green signals for Alexa Fluor 488. Scale bar = 50 µm. All data are shown as the mean ± SD, representative of three independent experiments. Statistical analyses of Foci and MFI of p-γH2AX were carried out using one-way ANOVA followed by Tukey’s multiple comparison test by GraphPad Prism software version 9.0.0. [file Image_1.jpeg]

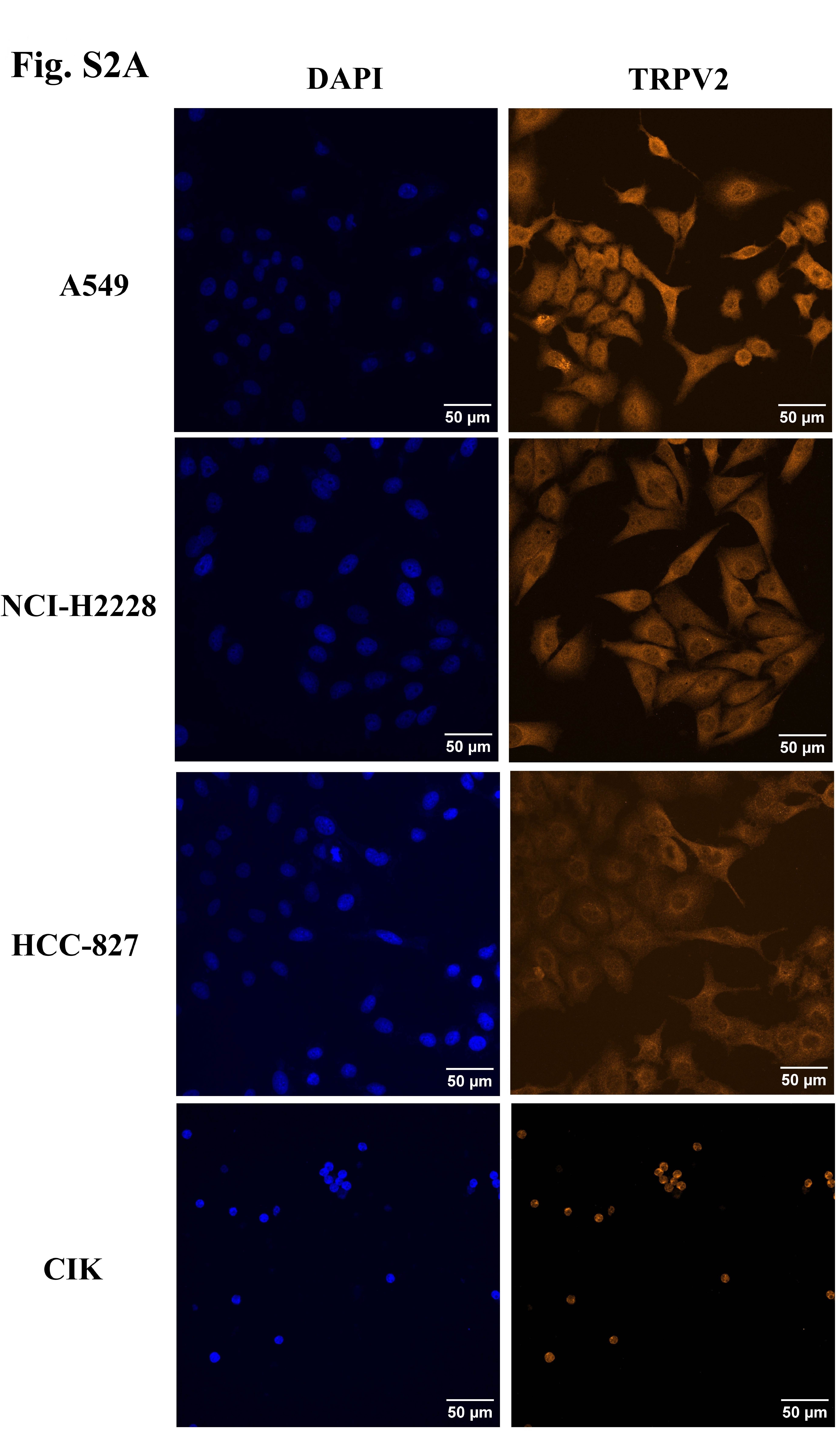

Supplement: Supplementary Figure 2 — Subcellular localization of TRPV2 in NSCLC cell lines and CIK cells detected by IHC and flow cytometry. (A) Cells were fixed and stained with NorthernLights™ NL557 with orange signals for TRPV2 and blue signals for nuclear DAPI. Fluorescent signals were captured by Visitron VisiScope Spinning Disk Confocal Microscopy and VisiVIEW® Image software. Scale bar = 50 µm. (B) CIK cells or NSCLC cells were stained with a Fixable Viability Zombie Aqua™ Dye exclude dead cells. After that, CIK cells were stained by APC anti-human CD3 antibody (clone OKT3), Brilliant Violet 421 anti-human CD8 antibody (clone RPA-T8), APC/cyanine7 anti-human CD4 antibody (clone RPA-T4), PE anti-human CD56 antibody (clone 5. 1 H11) at 4°C for 20 min. Afterwards, the cells were fixed and permeabilized using fixation buffer and intracellular staining permeabilization wash buffer. The blocking step was carried out in permeabilization wash buffer with 2% normal goat serum for 15 min. Cells were incubated with anti-TRPV2 antibody (1:100 dilution) at 4°C for 30 min. Subsequently, cells were washed and incubated with Alexa Fluor™ 488-labeled goat anti-rabbit IgG (H+L) (1:250 dilution) for 30 min. [file Image_2.jpeg]

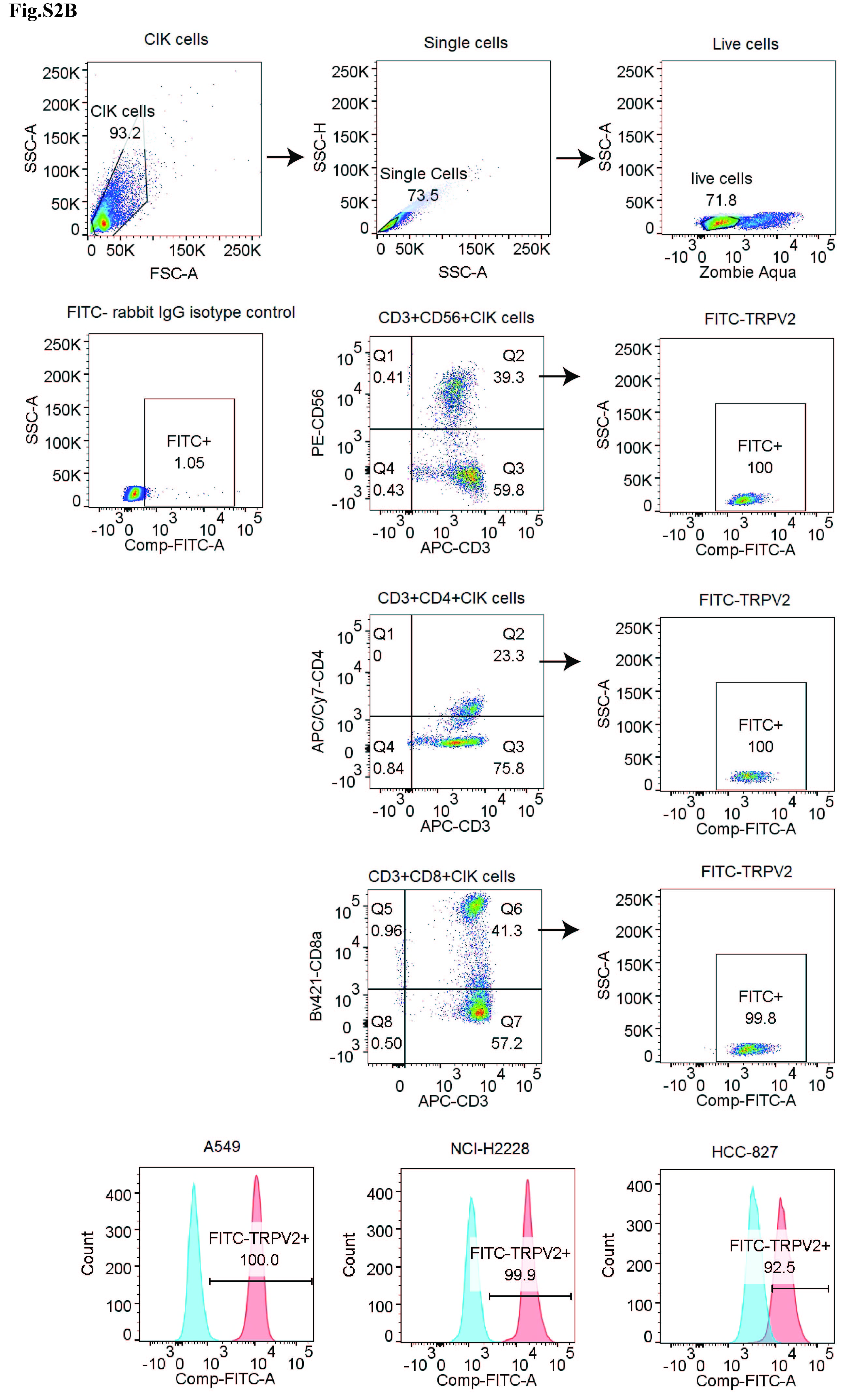

Supplement: Supplementary Figure 3 — The cytotoxicity of CBD on single NSCLC cells or single CIK cells. (A) The cytotoxicity of CBD on NSCLC cells and the cytotoxicity of CBD on CIK cells. 5x10^4^ CFSE-labeled NSCLC cells were incubated with CBD for 24 h. The cytotoxicity of CBD on CIK cells. 5x10^5^ CIK cells were incubated with CBD for 24 h. (B) Representative flow cytometry plots illustrated the typical gating strategy used to identify viable CIK cells after treatment with CBD. (C) Representative flow cytometry plots illustrated to identify viable CFSE-labeled NSCLC cells after treatment with CBD. *p < 0.05, **p < 0.01, ***p < 0.001, ****p < 0.0001 vs. Cells combined with DMSO were designed as a control. Statistical analysis was performed using a two-way ANOVA followed by Dunnett’s multiple comparison test by GraphPad Prism software version 9.0.0. Data are shown as the mean ± SD, representative of four independent experiments. CIK cells were derived from 4 donors. [file Image_3.jpeg]

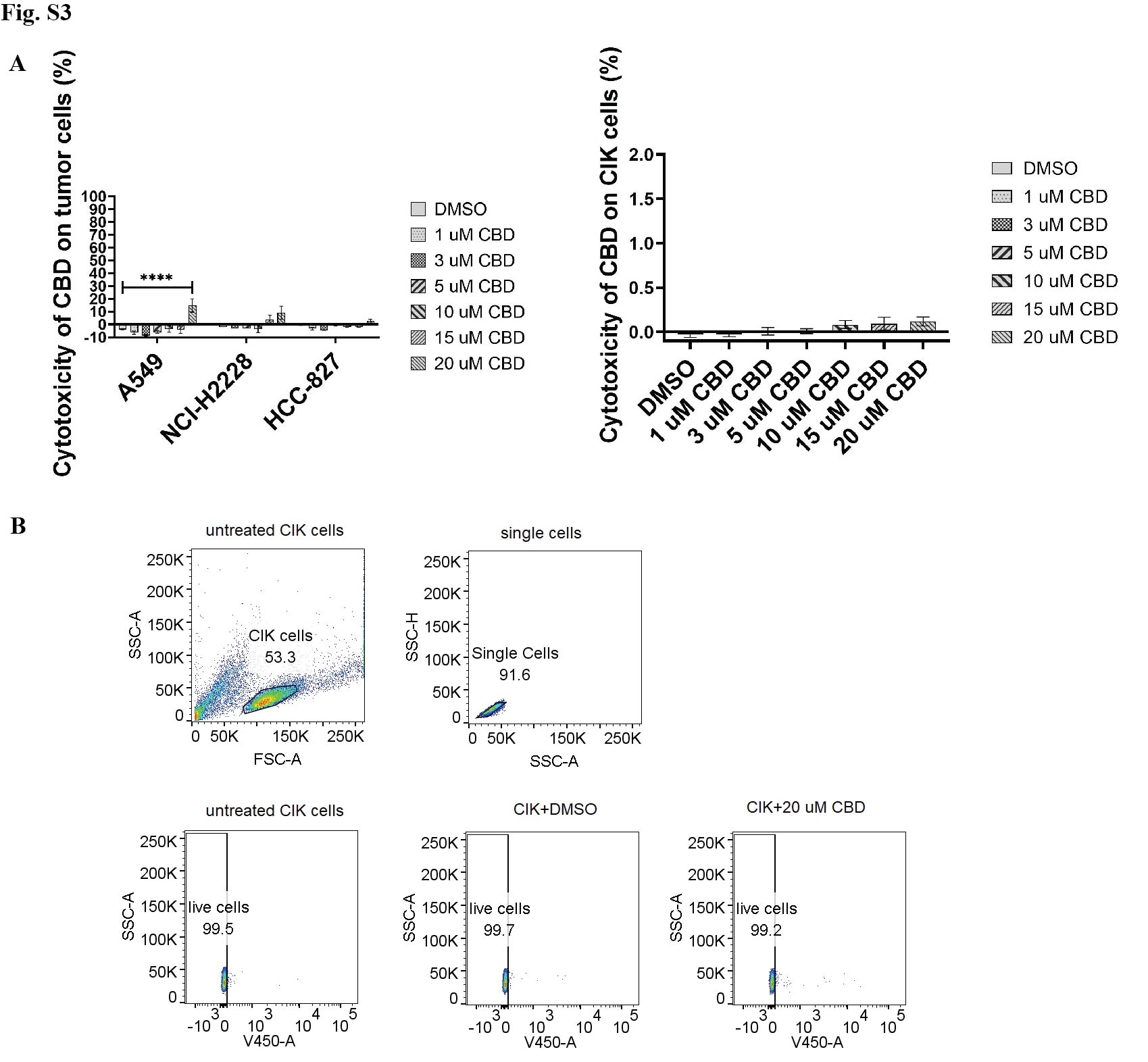

Supplement: Supplementary Figure 4 — (A) The surface expression of CD45RA and CD62L on CD3+CD8+CIK or CD3+CD4+ CIK cells after 24 h CBD treatment was detected by flow cytometry assessment. Naïve: CD45RA+CD62L+ T cells, CM: central memory T cells (CD45RA−CD62L+), EM: effector memory T cells (CD45RA−CD62L−), EMRA: terminal effector memory T cells (CD45RA+CD62L−). Statistical analysis was performed using a two-way ANOVA followed by Dunnett’s multiple comparison test by GraphPad Prism software version 9.0.0. CIK cells were derived from 4 donors. (B) PE-CD25 and PE-Cy7-CD69 gate strategies. The isotype PE-Cy7 IgG1k was a PE-Cy7-CD69 control and the isotype PE-IgG1k was a PE-CD25 control. [file Image_4.jpeg]

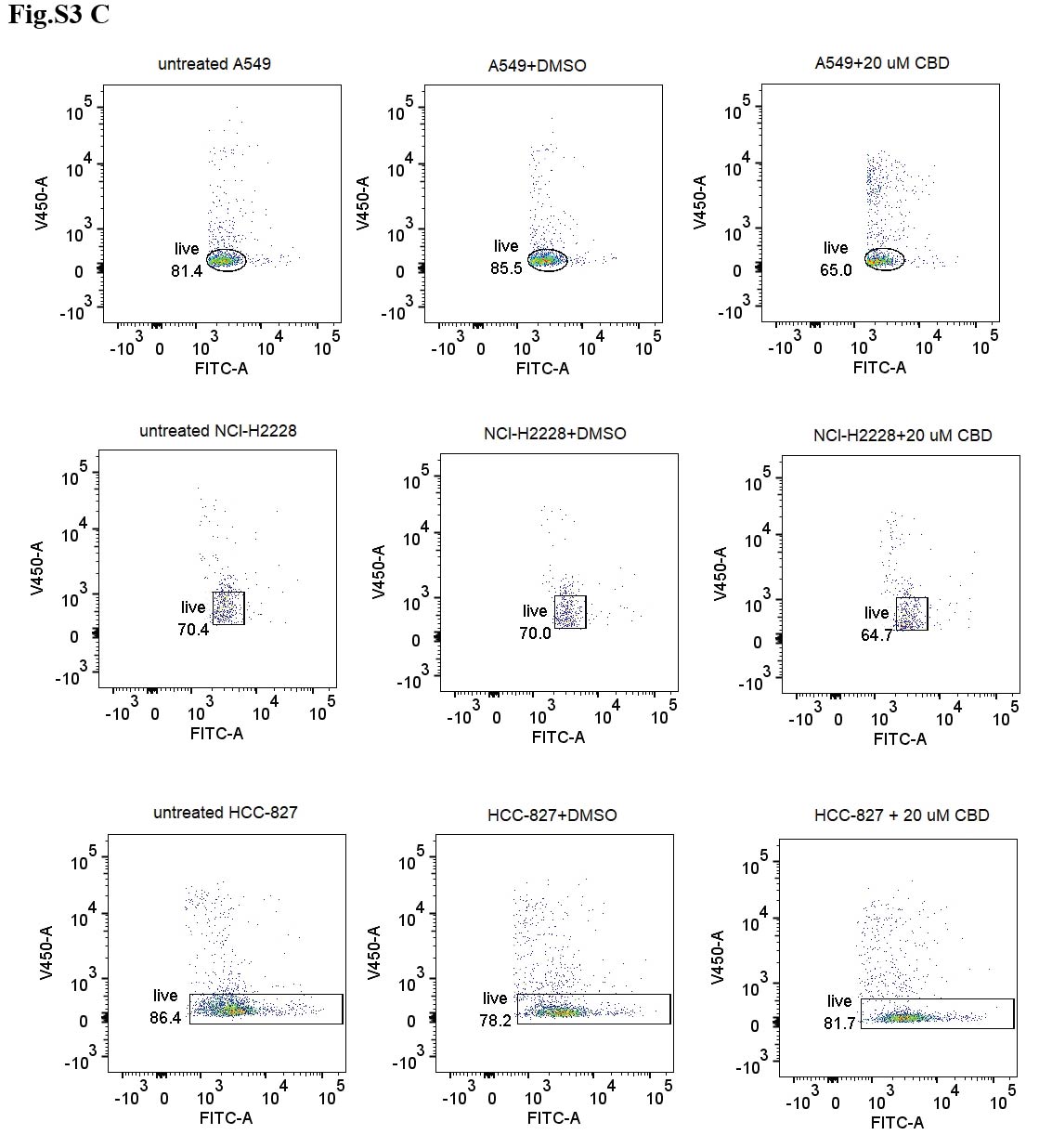

Supplement: Supplementary Figure 5 — The effects of ERK inhibitor FR180204 on the cytotoxicity of CBD-treated CIK cells against NSCLC cells. CIK cells were pretreated with 10 µM FR18024 for 2 h then cocultured with CFSE-labeled A549 (A), NCI-H2228 (B) and HCC-827 (C) at different CBD concentrations. E:T (effector-target) ratio =10:1. *p < 0.05, **p < 0.01, ***p < 0.001, ****p < 0.0001 vs. CIK combined DMSO targeting NSCLC cells was designed as a control. Statistical analysis was performed using a two-way ANOVA followed by Dunnett’s multiple comparison test by GraphPad Prism software version 9.0.0. Data are shown as the mean ± SD, representative of three independent experiments. CIK cells were derived from 3 donors. [file Image_5.jpeg]

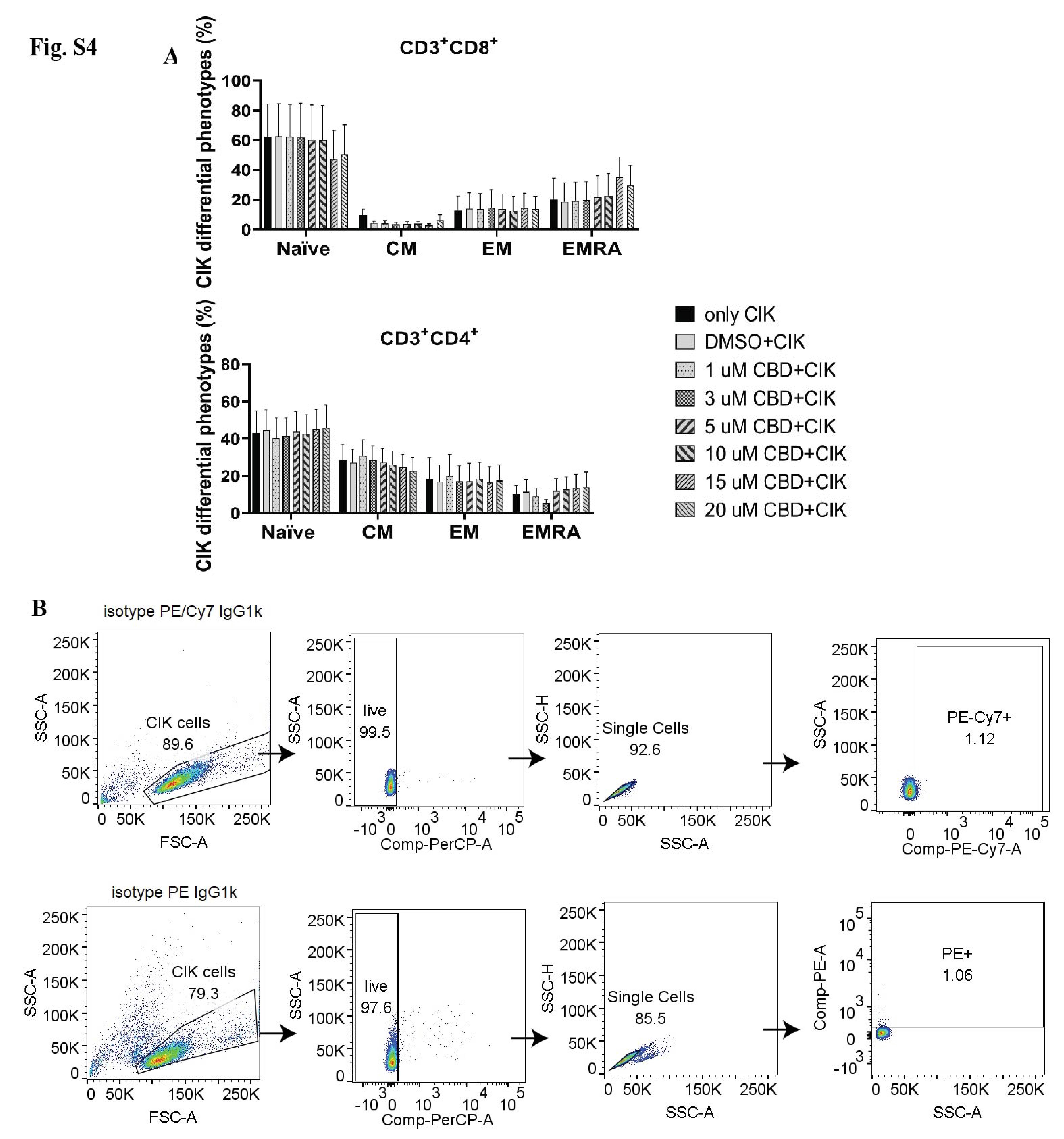

Supplement: Supplementary file 6 [file Image_6.jpeg]

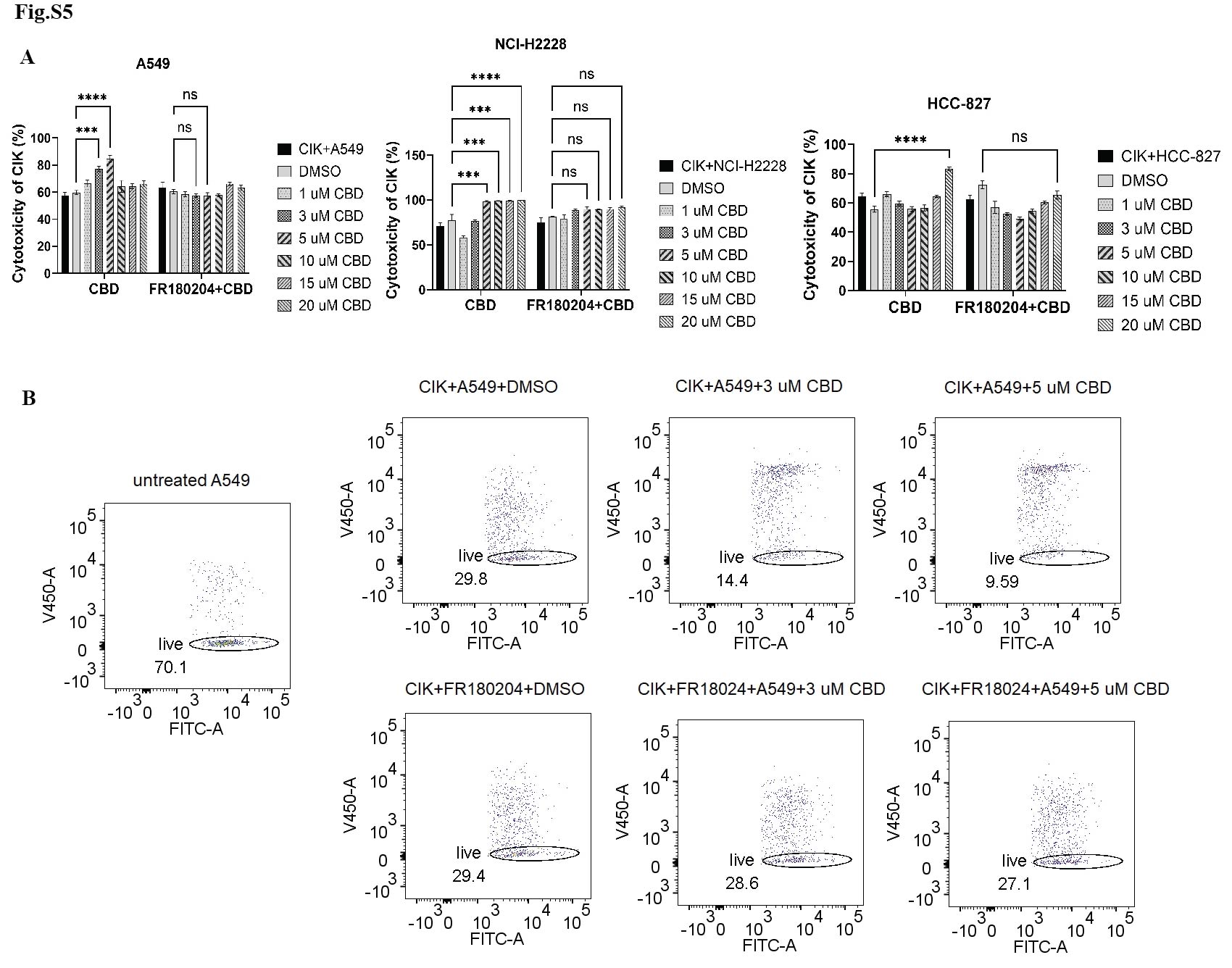

Supplement: Supplementary file 7 [file Image_7.jpeg]
